# Reconstructing an historical pollination syndrome: keel flowers

**DOI:** 10.1186/s12862-022-02003-y

**Published:** 2022-04-12

**Authors:** Deniz Aygören Uluer, Félix Forest, Scott Armbruster, Julie A. Hawkins

**Affiliations:** 1 School of Biological Sciences, Lyle Building, University of Reading, Whiteknights, Reading, Berkshire, RG6 6BX UK; 2 Royal Botanic Gardens, Kew, Richmond, Surrey, TW9 3DS UK; 3 School of Biological Science, University of Portsmouth, King Henry Building, King Henry I Street, Portsmouth, PO1 2DY UK

**Keywords:** Floral evolution, Pollination, Character reconstruction, Fabaceae

## Abstract

**Background:**

Keel flowers are bilaterally symmetrical, pentamerous flowers with three different petal types and reproductive organs enclosed by keel petals; generally there is also connation of floral parts such as stamens and keel petals. In this study, the evolution of keel flowers within the order Fabales is explored to investigate whether the establishment of this flower type within one of the species-rich families, the Fabaceae (Leguminosae), preceded and could have influenced the evolution of keel flowers in the Polygalaceae. We conducted molecular dating, and ancestral area and ancestral state analyses for a phylogeny constructed for 678 taxa using published *matK*, *rbcL* and *trnL* plastid gene regions.

**Results:**

We reveal the temporal and spatial origins of keel flowers and traits associated with pollinators, specifically floral symmetry, the presence or absence of a pentamerous corolla and three distinct petal types, the presence or absence of enclosed reproductive organs, androecium types, inflorescence types, inflorescence size, flower size, plant height and habit. Ancestral area reconstructions show that at the time keel flowers appeared in the Polygaleae, subfamily Papilionoideae of the Fabaceae was already distributed almost globally; at least eight clades of the Papilionoideae had keel flowers with a functional morphology broadly similar to the morphology of the first evolving Polygaleae flowers.

**Conclusions:**

The multiple origins of keel flowers within angiosperms likely represent convergence due to bee specialization, and therefore pollinator pressure. In the case of the Fabales, the first evolving keel flowers of Polygaleae have a functional morphology that corresponds with keel flowers of species of the Papilionoideae already present in the environment. These findings are consistent with the keel-flowered Polygaleae exploiting pollinators of keel-flowered Papilionoideae. The current study is the first to use ancestral reconstructions of traits associated with pollination to demonstrate that the multiple evolutionary origins of the keel flower pollinator syndrome in Fabales are consistent with, though do not prove, mimicry.

**Supplementary Information:**

The online version contains supplementary material available at 10.1186/s12862-022-02003-y.

## Background

Keel flowers sensu Westerkamp are bilaterally symmetrical (zygomorphic, monosymmetric), pentamerous flowers with three different petal types, with the reproductive organs enclosed by keel petals and generally with connation of floral parts such as stamens and keel petals [1–10]. Keel flowers are dominant in two species-rich lineages within Fabales, tribe Polygaleae Chodat (Polygalaceae Hoffmanns. & Link) and subfamily Papilionoideae L. (DC.) (Fabaceae Lindl.) [11, 12]. Indeed, this flower type is typical of subfamily Papilionoideae of Fabaceae, and the prevalence of keel flowers in Papilionoideae has prompted some to refer to them as papilionate flowers.

Fabaceae is the third largest angiosperm family with approximately 765 genera and 19,500 species [13–16]. The keel-flowered subfamily, Papilionoideae, includes almost 72% of Fabaceae species, ca. 14,000 species in 504 genera [8, 14, 15]. Similarly, within Polygalaceae (with approximately 1000 species in 20 genera), tribe Polygaleae holds 80% of the species richness of the family with ca. 800 spp. [1, 5, 17]. In the Fabaceae, keel flowers are also found outside of Papilionoideae, in Cercidoideae, Dialioideae and Caesalpinioideae: *Cercis*, *Poeppigia procera* C. Presl and *Peltophorum* Vogel (Benth.) [18, 19]. Other, unrelated families including species with keel flowers are the Ranunculaceae, Onagraceae, Sapindaceae, Trigoniaceae, Geraniaceae, Tropaeolaceae, Solanaceae, Acanthaceae and Commelinaceae, but they have fewer keel-flowered species than either Fabaceae or Polygalaceae [9].

In general, the evolution of keel flowers within Fabaceae, Polygalaceae and different clades of angiosperms has been attributed to bees [9, 20–22], but particularly to skilled and strong bees [9, 18, 20, 21, 23–25]. Less commonly, large and brightly coloured flowers may be bird-pollinated (e.g., *Mucuna* Adans., *Erythrina* L.), or butterfly-pollinated (e.g., *Berlinia grandiflora* Vahl Hutch. & Dalziel), and a few keel-flowered species with specific scents are beetle (e.g., *Aotus lanigera* A. Cunn. ex Benth.) or fly-pollinated (e.g., *Apios americana* Medik.) [18, 26, 27]. Specific adaptations of keel flowers are associated with bee pollination. For example, hiding pollen inside keel flowers protected by a tripping mechanism may limit pollen loss associated with pollen theft [9, 10, 18, 19, 21, 28]. Furthermore, many Papilionoideae flowers exhibit different primary and secondary pollination mechanisms such as valvular, pump, explosive and brush mechanisms, which also ensure accuracy and efficiency of pollen deposition and so limit the pollen waste ([29] and references therein). In these ways, pollen is hidden in the deepest part of the flower, and pollen is transmitted to, for example, a bee’s head, or above the insertions of the legs or wings, so pollen cannot be easily removed during grooming [9, 10, 30, 31]. This specific position of the pollen (i.e., location of the pollen on different bee species, such as back of the head, under the mandible or on inner side of mandible) is also another precaution against non-pollinator visitors [30]. Thus, the evolution of keel flowers has been referred to as an adaptive response to bees; the keel flowers evolved not just to attract bees but also to protect the pollen from bee robbery [9, 18, 22, 31, 32]. The independent evolution of the keel flower syndrome in the Fabales is likely to have secured pollination and promoted cross-pollination of keel-flowered species [33, 34].

Previously, the morphology and development of keel flowers of two species-rich clades of Fabales, Papilionoideae and Polygaleae, have been compared (e.g., [1, 17, 22, 35]). In both clades, keel flowers are at least superficially similar, they both are 5-cyclic and 5-merous, and consist of three parts, a standard for visual attraction, two wings as a landing platform and a keel to conceal the pollen from pollinators [8, 9, 18, 22, 35]. However, while the functions of these parts are the same in the two groups of Fabales, the developmental origins are different. The standard (flag) consists of a single median petal in Fabaceae but is composed of two lateral sepals in Polygalaceae. The wings are formed by two petals in Papilionoideae but consist of two petaloid lateral sepals in Polygalaceae [22]. One or two fused lower lateral petals serve as the keel in legumes, but the keel comprises one median petal in Polygalaceae, since Polygalaceae keel flowers consist of five petals, only three of which are fully developed, and the abaxial one forming an asymmetric keel [5, 22, 36–38]. Keel flowers of Papilionoideae have ten stamen filaments and a single carpel, but Polygalaceae keel flowers have eight filaments and a syncarpous gynoecium which consists of two carpels [22, 35]. Thus, keel flowers in the two families represent a superficial functional and morphological convergence, rather than a homologous similarity [1, 17, 22, 35].

Although the flowers of these two Fabales lineages are not homologous, their striking similarity has led some authors to propose that this shared similarity is more than convergance on a floral syndrome. Bello et al. [17] proposed that the rapid diversification of the tribe Polygaleae, previously documented by Forest et al. [39], may have been prompted “because pollinators of pre-existing papilionoid legume flowers were immediate and effective pollinators of the later-evolving papilionoid flowers of Polygaleae”. Indeed, Bello et al. [17] were not the first to propose such a scenario. Pseudo-papilionoid flowers of *Cercis* L. (sensu Polhill et al. [19]) have some similarities with Papilionoideae keel flowers such as a bilaterally symmetrical corolla, three different petal types, and enclosed stamens and gynoecium, prompting Tucker [7] to hypothesize a mimicry relationship between *Cercis* keel flowers and Papilionoideae flowers. Tucker [7] supposed that *Cercis* likely evolved in an environment where keel flowers were already present. However, whether the later-evolving keel flowers benefitted from pollinators familiar with the keel-flowers of earlier-evolving clades is an open question.

To meet the criteria which evidence floral mimicry, species should share a common pollinator which freely moves between two taxa, share display traits apparent to the pollinators (e.g., colour, UV patterns, nectar, scent and size), there should be a reproductive benefit to one or both species, the species should have areas of sympatry, overlapping flowering phenology, and maybe most importantly the mimic should be more successful in terms of reproduction because of its resemblance to the model, so the mimic receives more visits when it co-occurs with the model [40–43]. Aside from numerous examples of mimicry involving the Orchidaceae (e.g., [44]), floral mimicry is evidenced for angiosperm and gymnosperm beetle pollinated plants [45] and Turneraceae and Malvaceae flowers [40], with the study of oil-offering plants the first to scrutinise putative floral mimicry in deep evolutionary time [46]. Identifying older and younger oil-offering clades, Renner and Schaefer [46] suggested that ancestral reconstructions were consistent with a gradual niche broadening, but that attributing each later appearance to mimicry would depend on studies of pollinator behavior. For inferences based on phylogenetic reconstructions, in the absence of observations of pollinator behaviour, we therefore refer to findings consistent with a mimicry, but we cannot provide evidence of mimicry from evolutionary reconstructions alone. However, though this was not the case, we could have refuted mimicry if keel flowers of younger clades did not share pollination syndromes or were not sympatric with species in existing clades.

The aim of the current study is to determine whether evolution of the keel flowers of Polygaleae and Papilionoideae are consistent with a mutualistic relationship by characterising the early evolution of the keel flowers of these two species-rich clades. Specifically, we set out to confirm whether keel flowers appeared first in Papilionoideae, whether the keel-flowered Papilionoideae were likely to have been present in the geographical areas where the keel-flowered Polygaleae first appeared, and whether the functional morphology of the keeled Papilionoideae flowers was broadly similar to the morphology of the first evolving Polygaleae flowers. If these criteria are met, though not proving mutualism, this would be consistent with a mutualistic relationship, with Polygaleae flowers benefitting from existing Papilionoideae pollinators due to their resemblance to pre-existing Papilionoideae keel flowers.

## Results

### Molecular dating analysis

**Fig. 1:**
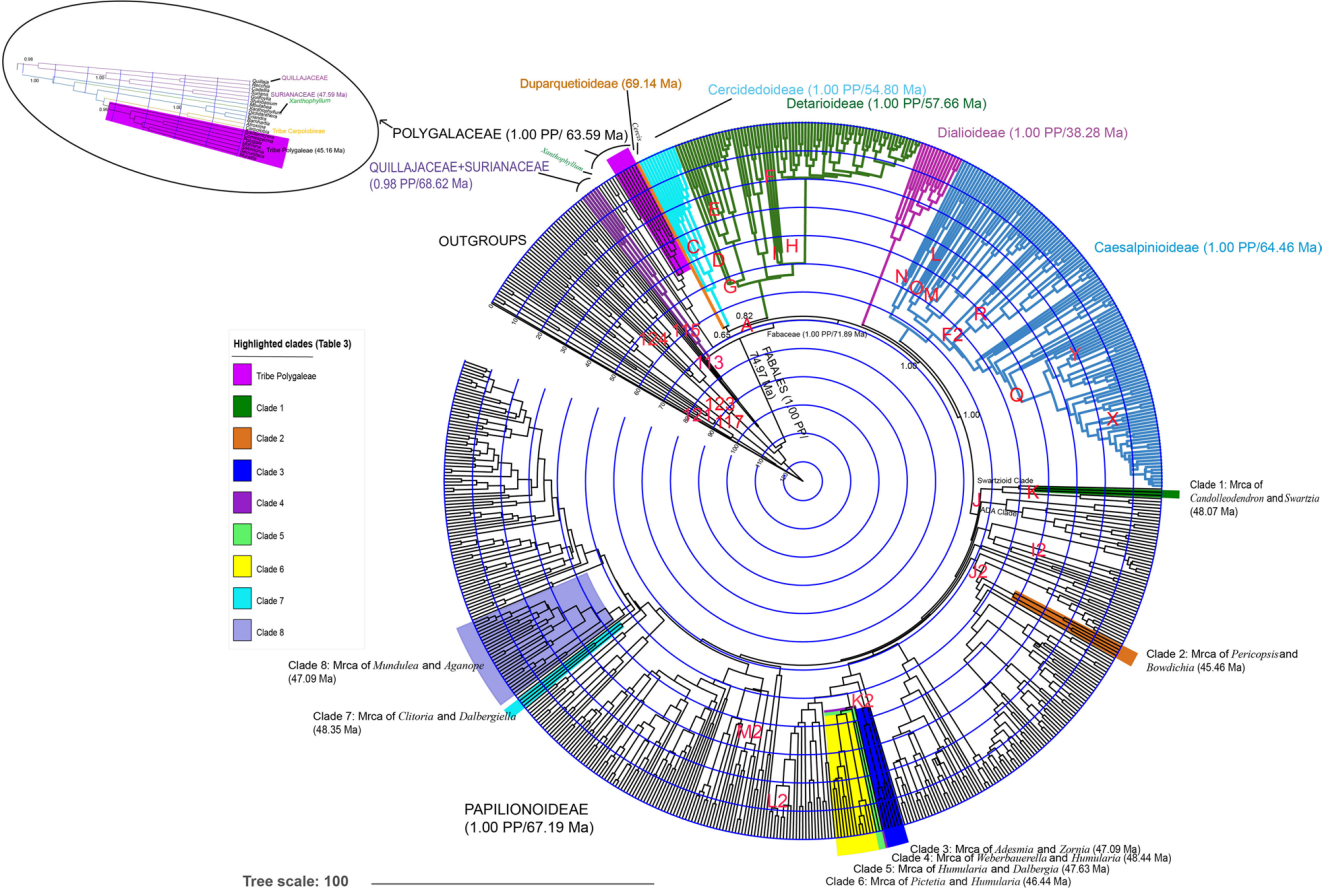
The origins of Papilionoideae clades (Clade 1–8) which evolved during 49–45.16 Ma and the origin of the evolution of keel flowers within Polygalaceae. Posterior probabilities for the key nodes are indicated. Four Fabales families, six Fabaceae subfamilies, *Cercis* and *Xanthophyllum* are indicated. Standard error bars were excluded from the figure for clearer presentation. Letters and numbers in red correspond to the calibration points. Scale bar in million years

The divergence time analysis (Additional file 1: S1) generated a ((Fabaceae+Polygalaceae) (Surianaceae+Quillajaceae)) topology within monophyletic Fabales (1.00 PP). Within Polygalaceae, tribe Moutabeae was not monophyletic (Fig. 1). However, both tribe Polygaleae and tribe Carpolobieae were strongly monophyletic (0.96 and 1.00 PP, respectively). On the other hand, a (((Papilionoideae+Caesalpinioideae) Dialioideae) (Detarioideae (Duparquetioideae+Cercidoideae))) topology is estimated within Fabaceae. *Duparquetia* Baill. (Duparquetioideae) was sister to monophyletic Cercidoideae (1.00 PP) with posterior probability of only 0.65. Monophyletic Detarioideae (1.00 PP) was sister to this clade with moderate support (0.82 PP). Monophyletic Caesalpinioideae (1.00 PP) was sister to monophyletic Papilionoideae (1.00 PP) with posterior probability of 1.00, and monophyletic Dialioideae (1.00 PP) was sister to this clade (1.00 PP).

The crown age of Fabales is estimated to be at least 74.97 Ma (95% HPD 69.3–76.7); the (Surianaceae+Quillajaceae) crown node as 68.62 Ma (95% HPD 50.2–73.9); Surianaceae crown node as 47.59 Ma (95% HPD 33.2–53.1); Fabaceae crown node as 71.89 Ma (95% HPD 67.9–69.3), subfamily Papilionoideae crown node as 67.19 Ma (95% HPD 62.5–64.9); Polygalaceae family crown node as 63.59 Ma (95% HPD 58.2–62.7); and 45.16 Ma (95% HPD 38.8–44.7) for the crown node of tribe Polygaleae. Additionally, the molecular dating analyses yielded a 54.80 Ma (95% HPD 55.1–48.1) crown age for the crown Cercidoideae node, 57.66 Ma (95% HPD 55.6–53.1) for the crown Detarioideae node, 38.28 Ma (95% HPD 44.7–27.1) for the crown Dialioideae node, 64.46 Ma (95% HPD 64.1–58.2) for the crown Caesalpinioideae node, and 69.14 Ma (95% HPD 68.5–63.3) for Duparquetioideae.

The first node with keel flowers in the Polygalaceae was 46.98–45.16 Ma (crown age of tribe Polygaleae). The evolution of keel flowers coincided with the evolution of the keel flower tribe, Polygaleae (99.6%) (Table 1).

**Table 1:** Results of ancestral area, age, ancestral flower type, ancestral floral symmetry, the presence or absence of a pentamerous corolla (petals+sepals in Polygalaceae), the presence or absence of three distinct petal types (petals+sepals in Polygalaceae), the presence or absence of enclosed reproductive organs, ancestral androecium type, ancestral inflorescence type, ancestral floral size, ancestral height and habit analyses for tribe Polygaleae, and clades 1–8 of Papilionoideae

Name of the clade	Geographic origin	Age (~ my)	Ancestral floral type	Floral symmetry	Pentamerous petals (+ sepals)	Three distinct petal (+ sepal) types	Enclosed reproductive organs	Androecium type	Inflorescence type	Flower size (mm)	Height (m)	Habit
Tribe Polygaleae (Polygalaceae)	**67% G** 18% EG 14% E	**45.16** (~ 38.8–44.7)	**99.6% A** (99.3–99.9%)	21% A (20.2–21.5%) **79% B** (78.5–79.1%)	**63% A** (62.7–63.2%) 37% B (36.9–37.3%)	**89.6% A** (89.3–89.8%) 10.2% C (10–10.5%)	**99.9% A** (99.7–100%)	2% A (1.6–2.4%) **98% B** (97.6–98.4%)	**81% A** (79.7–81.8%) 1% B (1.2–1.5%) 18% C (16.9–18.9%)	**2.87–10.5** (2.29–11.7)	**0.76–9.1** (0.45–9.8)	6% A (5.5–6.1%) **41% B** (40.5–41.8%) **53% C** (52.2–53.9%)
Clade 1 (Mrca of *Candolleodendron* and *Swartzia*	**100% G**	**48.07** (~ 44.0–46.0)	**99.9% B** (99.9–100%)	6% A (6.1–6.3%) **94% B** (93.7–94%)	1% A (0.5–0.6%) **99% B** (99.3–99.4%)	**94% B** (93.9–94.1%) 6% C (5.9–6.1%)	**100% A** (100%-100%)	**100% A** (100%-100%)	**97% A** (96.9–97.3%) 1% B (0.5–0.6%) 2% C (2.1–2.5%)	X	**2.1–22.8** (1.9–23.3)	**98% A** (98.1–98.2%) 1% B (0.9–1%) 1% C (0.8–0.9%)
Clade 2 (Mrca of *Pericopsis* and *Bowdichia*)	**66% G** 34% EG	**45.46** (~ 30.1–53.8)	**99.9% A** (99.8–100%)	2% A (1.9–2%) **98% B** (98–98.1%)	**96% A** (96.1–96.3%) 4% B (3.7–3.9%)	41% A (39.6–42.3%) **58.2% C** (56.9–59.5%)	**99.8% A** (99.7–99.9%)	**99.9% A** (99.9–100%)	**97% A** (96.5–97.4%) 3% C (2.5–3.4%)	**11.6–24.5** (11.2–25.4)	**8.1–34** (7.9–34.4)	**95% A** (94.6–95.6%) 1% B (0.4–0.5%) 1% C (0.3–0.5%)
Clade 3 (Mrca of *Adesmia* and *Zornia*)	**100% G**	**47.09** (~ 36.6–51.5)	**99.9% A** (99.9–100%)	24% A (23.3–23.7%) **76% B** (76.3–76.7%)	**61% A** (60.7–61%) 39% B (39–39.3%)	**81.1% A** (80.9–81.3%) 1.3% B (1.2–1.3%) 17.6% C (17.4–17.8%)	**99.9% A** (99.9–100%)	4% A (4.3–4.4%) **96% B** (95.6–95.7%)	**81% A** (79.7–81.3%) 6% B (5.4–6.1%) 1% C (1.3–1.4%)	**8.3–16** (7.7–17.5)	**2.9–10.8** (2.5–11.7)	29% A (28.3–29.7%) 28% B (27.3–28.6%) **43% C** (42.3–43.9%)
Clade 4 (Mrca of *Weberbauerella* and *Humularia*)	**100% G**	**48.44** (~ 42.8–47.3)	**99.9% A** (99.9–100%)	7% A (6.4–6.6%) **93% B** (93.4–93.6%)	**78% A** (77.5–78%) 22% B (22–22.5%)	**95.4% A** (95.2–95.6%) 4.5% C (4.3–4.7%)	**99.9% A** (99.9–100%)	**99.9% B** (99.8–100%)	**91% A** (90.8–91.9%) 2% B (1.7–2.1%) 7% C (6.2–7.3%)	**8.3–20** (7.9–21.1)	**3.2–19.6** (2.9–20.2)	3% A (3.1–3.2%) **95% B** (94.8–95%) 2% C (1.9–2%)
Clade 5 (Mrca of *Humularia* and *Dalbergia*)	**100% G**	**47.63** (~ 42.8–47.3)	**99.9% A** (99.9–100%)	7% A (6.6–6.7%) **93% B** (93.1–93.4%)	**78% A** (77.5–78%) 22% B (22–22.5%)	**93.3% A** (93–93.6%) 6.6% C (6.4–6.9%)	**99.9% A** (99.9–100%)	**99.9% B** (99.8–100%)	**89% A** (88.2–89.5%) 3% B (2.5–2.9%) 8% C (7.9–9%)	**8–18.74** (7.5–19.8)	**2.9–19** (2.7–19.5)	4% A (4.1–4.4%) **93% B** (92.9–93.3%) 3% C (2.5–2.7%)
Clade 6 (Mrca of *Pictetia* and *Humularia*)	**100% G**	**46.44** (~ 38.4–46.6)	**99.9% A** (99.8–100%)	16% A (15.3–15.7%) **84% B** (84.3–84.7%)	**67% A** (66.7–67.2%) 33% B (32.8–33.3%)	**83.6% A** (83.4–83.8%) 15.8% C (15.6–16.1%)	**99.9% A** (99.9–100%)	**99.5% B** (95.4–95.6%)	**85% A** (83.7–85.4%) 2% B (2.5–2.9%) 13% C (11.9–13.5%)	**8.2–20.5** (7.7–21.6)	**2.8–19.1** (2.5–19.7)	4% A (4–4.3%) **84% B** (83.2–84.1%) 12% C (11.7–12.3%)
Clade 7 (Mrca of *Clitoria* and *Dalbergiella*)	**48% B** **45% G**	**48.35** (~ 38.5–50.4)	**99.8% A** (99.6–100%)	27% A (26.5–26.9%) **73% B** (73.1–73.5%)	**60% A** (60.3–60.5%) 40% B (39.5–39.7%)	**77.5% A** (77.3–77.7%) 20.5% C (20.4–20.7%)	**99.9% A** (99.9–100%)	1% A (1.4–1.5%) **99% B** (98.5–98.6%)	**43% A** (43.1–43.3%) **44% B** (43.7–44.1%) 13% C (13.1–13.4)	**10.3–25.4** (9.7–26.8)	**4.2–16.6** (3.9–17.4)	**46% A** (45.9–46.2%) **41%B** (40.4–40.8%) 13%C (13.1–13.5%)
Clade 8 (Mrca of *Mundulea* and *Aganope*)	**35% BG** **29% B** **22% G**	**47.09** (~ 40.1–46.5)	**98% A** (98–98.3%) 2% B (1.8–2%)	25% A (23.6–26.6%) **75% B** (73.4–76.4%)	**65% A** (64–66.1%) 35% B (33.9–36%)	**87.4% A** (86.5–88.3%) 12.3% C (11.5–13.2%)	**99.6% A** (99.4–99.8%)	**90% A** (88.8–90.9%) 10% B (9.1–11.2%)	**90% A** (89.6–91.1%) 4% B (3.1–4%) 6% C (5.6–6.6%)	**10–20.3** (9.6–21.4)	**7.4–17.4** (7.2–17.9)	**63% A** (61.7–64.4%) 23% B (21.8–23.8%) 14% C (13.6–14.9%)

In each column, the bold value indicates the highest probability for each character. Decimals are rounded to the nearest whole number to avoid fractional points. Possibilities less than 1% are not included, and the ancestral area analyses up to three possible origins are reported. Nodes that do not fulfill the criteria of a mimicry scenario are shaded. Crosses (X) indicate that the corresponding analysis was not employed due to lack of information. Ages (My) are mean and node heights from HPD intervals at 95% (upper and lower) and 95% confidence intervals for the discrete and continuous characters are provided within parantheses. For the ancestral flower size and plant height analyses, both the smallest and largest sizes are indicated. Geographic area abbreviations: A: Eurasia; B: Africa; C: Madagascar; E: Australia including New Guinea, New Caledonia and New Zealand; F: North America; G: South America including Central America and H: the Indian subcontinent [47]. Other abbreviations are explained in Table 3

*Mrca* most recent common ancestor, *my* million years, *mm* millimeter, *m* meter

Based on the reconstructions of the timing of keel flower origin in Polygalaceae (46, 98–45, 16 Ma), we selected the Papilionoideae nodes which were extant in South America at that time to test whether a mimicry between Polygalaceae and Papilionoideae was plausible. To be conservative, we expanded the time frame up to 49 Ma. In this logic, there were 16 clades in total. However, out of these 16 nodes, one originated between ~ 49 and 45 and could not be included here due to lack of information. We also excluded seven clades due to their geographic distribution (not included in Table 1). For the eight remaining relevant clades, the ancestral area and ancestral state for each of the 11 pollination syndrome characters were reconstructed. These clades are shown in Fig. 1, listed in Appendix 2, and the ages of these nodes are reported in Table 1.

### Ancestral area analyses

The Lagrange analyses indicated a possible South American origin for the keel flowered Polygalaceae (67% G, 18% EG, 14%E) (Table 1, Additional file 2: S2). For the eight Papilionoideae clades hypothesized, ancestral areas are listed in Table 1.

Additionally, the Lagrange analyses support a South American origin for the subfamily Papilionoideae (100%) and Africa+South American origin for the Fabaceae (75% BG, 14% BEG, 11% G). A South American origin was also suggested by Lagrange analysis for the origin of Fabales with low support (30% G, 29% BEG, 20% BG).

### Ancestral state analyses

The ancestral floral type for Polygalaceae was non-keeled (92%), whilst the ancestral flower type of tribe Polygaleae was keeled (99.6%) with three distinct petal+sepal types (89.6%), enclosed reproductive organs (99.9%), fused stamens (98%), probably pentamerous petals+sepals (63%) and bilateral symmetry (79%) (Table 1). The most recent common ancestor (MRCA) of tribe Polygaleae most possibly had vertical inflorescences (e.g., raceme, panicle, spike and thyrse) (81%), and a small habit (e.g., herb, 53%; however, the possibility of a medium-sized habit was also high at 41%). The ancestral floral size of tribe Polygaleae was between 2.9 and 10.5 mm, and ancestral plant height was between 76 cm and about 9.1 m.

There were eight clades of Papilionoideae with ancestral morphologies that might have allowed pollinators to move freely between the flowers of these and tribe Polygaleae flowers. These clades were clades 2–8 (Table 1), based on shared pollination syndrome characters. Where characters were not a perfect match, but differences were not likely to significantly impact pollinators, we considered shared pollinators a possibility. For example, ancestral reconstruction of clade 2 suggested these plants were of similar height to the ancestors of tribe Polygaleae, so despite the different habits we considered it possible that ancestors of this clade shared pollinators with tribe Polygaleae. Similarly, ancestral reconstructions of free stamens in clades 2 and 8 were not considered to have significant impact on pollinator behavior because other ancestral floral characteristics of these two clades match the ancestral flowers of tribe Polygaleae, and keel flowers with free stamens still exist in Papilonideae (e.g., *Bolusanthus* and *Baptisia*).

## Discussion

To determine whether the first-evolving Polygalaceae keel flowers were functionally similar to existing Papilionoideae keel flowers, and might have evolved in an environment where functionally similar Papilionoideae keel flowers were present, we carried out temporal, spatial and trait analyses.

The divergence-time analysis was congruent with previous studies (e.g., [12, 39]), with short internal branches of Fabales reflecting the rapid radiation already highlighted for this clade [12, 48, 49]. The divergence-time analysis also showed that keel flowers in crown Papilionoideae 67.19 Ma (95% HPD 62.5–64.9) evolved before the origin of Polygalaceae (63.59 Ma, 95% HPD 62.7–58.2 Ma) and tribe Polygaleae (45.16, 95% HPD 44.7–38.8 Ma), even if we accept *Xanthophyllum* as polymorphic in terms of the presence or absence of keel-flowers [50]. Similarly, even accepting a keel flowered ancestor for Polygalaceae (*Moutabea* Aubl. excluded), the evolution of keel flowers within crown Papilionoideae was still earlier than in the crown Polygalaceae (56.87 Ma, 95% HPD 62.7–58.2 Ma). We are confident that keel flowers within Papilionoideae evolved many millions of years (22.03–10.32 Ma) before the evolution of keel flowers in Polygalaceae.

Our ancestral area reconstructions show that at the time keel flowers appeared in the Polygaleae, the Papilionoideae was distributed almost globally. There were eight clades in South America at the time that the keel-flowered Polygaleae originated in this continent. Trait analyses show that at least seven of these clades are consistent with a mutualistic relationship. In other words, at least for these seven nodes, the functional morphology of the keeled Papilionoideae flowers was broadly similar to the morphology of the first evolving Polygaleae flowers. Thus, Polygaleae flowers might have benefited from existing Papilionoideae pollinators that were already visitors to Papilionoideae keel flowers.

When pollinators are available, “a plant should specialize on the most effective and/or most abundant pollinator” [51, 52]. In this case, if Papilionoideae keel flowers’ pollinators are readily available, exploratory visits by these effective and/or abundant pollinators to Polygaleae flowers resembling Papilionoideae keel flowers might have been the first stage of this ancient mimicry [46]. In this case, if the Polygaleae keel flowers were rewardless, this could be a type of Batesian mimicry; however, if the Polygaeae keel flowers possessed abundant amount of reward, and this mutualism could be beneficial to both model and the mimic, this could be a Müllerian mimicry. Yet, whether Polygaleae keel flowers were more or less rewarding than the keel flowers of Papilionoideae is an open question. We suppose that Polygalaeae exploited existing Papilionoideae pollinators, but co-flowering might have increased the pollination rate of both Papilionoideae and Polygaleae keel flowers at that time [53]. However, since we are reconstructing a scenario that occured millions of years ago, it is almost impossible to determine the community interactions that occurred among existing plants and their pollinators. Without knowing these community interactions robust evidence for mimicry is elusive, because in a mimicry scenario the model is abundant in the environment, but the mimic is in low densities, while in a convergent evolution scenario both mimic and model are found in similar densities [40].

Pollinator shifts can cause diversification [54, 55], and a shift to long-tongued bees might have been one key innovation associated with the diversification rate shift already documented for the stem lineage of Polygaleae [39]. Indeed, pollinator shifts account for ca. 25% of documented divergence events in the Orchidaceae plus ca. 25% of other angiosperms [54, 56]. Subsequent to the origin of keel flowers, successive ecological opportunities to colonize new habitats [54], expanding the geographic and niche range [57, 58] has likely accompanied the diversification of the keel-flowered lineages. For the subfamily Papilionoideae, on the other hand, radiations in multiple lineages have driven increases in species richness [59]. In the current study, we did not perform any diversification rate analyses for Papilionoideae, and whether keel flowers are key innovations in this subfamily remains an open question.

Complex flowers, such as keel and bilabiate flowers (i.e., dorsiventral blossoms sensu [9, 10], are hypothesized to have evolved to hide protein rich pollen from bees by blocking the entrance of the flower, and at the same time, to ensure pollination by attaching pollen to either the dorsal (bilabiate or lip blossoms) or ventral (most keel blossoms) side of bees [9, 10, 20, 30, 51, 54]. Westerkamp [9] and Westerkamp & Claßen-Bockhoff [10] stated that, in terms of pollination, while the evolution of keel flowers is much more effective, keel and lip flowers are fundamentally the same. While lip flowers evolved in at least 38 angiosperm families (including Fabaceae), keel flowers evolved at least 16 times within 10 different orders independently within angiosperms. However, excepting Trigoniaceae and Fumarioideae, there are fewer species of keel flowers in angiosperm families other than in Fabales [9, 22]. Some genera or species of these other families have flowers with notable morphological similarities to legume keel flowers, such as *Pelargonium rapaceum* (L.) L’Hér. (Geraniaceae), *Calceolaria* L. (Lamiales) and *Monopsis lutea* (L.) Urb. (Campanulaceae) with enclosed reproductive organs and standard(s); *Schizanthus* Ruiz & Pav. (Solanaceae) with a standard, wings and keel petals [7, 9, 18, 19, 60]. *Collinsia* Nutt. (Lamiales) with a standard, wings, a keel and hidden reproductive organs, has greatest similarities with Papilionoideae and Polygaleae keel flowers [9, 60–63]. In *Aconitum* L., pollen is also deposited on the ventral side of the pollinator during pollination. In *Corydalis cava* (L.) Schweigg. & Körte on the other hand, lateral petals enclose the reproductive organs to hide pollen from bumblebees, as in Papilionoideae keel flowers [28]. Both *Hyptis* Jacq. (Lamiaceae) and *Levenhookia* R.Br. (Stylidiaceae) have spring-loaded keels which cause explosive release of fertile parts when triggered by pollinators.

The similarities of these keel flowers have been attributed to convergence due to bee specialization, and therefore, pollinator pressure [9, 54, 64, 65]. In this case, not only the morphological characters of keel flowers but also the choices of pollinators (e.g., exact location of pollen on bees’ body where it cannot be removed easily, enclosed reproductive organs which supports the minimum loss of pollen by keeping it away from non-functional visitors, visual clues such as bilateral symmetry, a standard for visual attraction, a landing platform and a standard for visual impact) might have canalized the convergent evolution of keel flowers within unrelated angiosperm clades. Here, for example, keel flowered *Collinsia heterophylla* Graham (Lamiales), *Aconitum napellus* subsp. *lusitanicum* Rouy (Ranunculales) and *Corydalis cava* (Ranunculales) are also pollinated by similar pollinators as the keel flowers of Papilionoideae [60–62, 66–69]. Keel flowers might have evolved to host these efficient pollinators (strong bees or long-tongued bees of Apidae and Megachilidae families) [63, 67, 70]. Indeed, Ruxton and Schaefer [71] suggested, instead of mimicry, that convergent evolution (i.e., pollination syndromes sensu Faegri andVan der Pijl [25]) driven by shared pollinators could be a more plausible explanation not only for the evolution of keel blossoms within unrelated lineages of angiosperms [9, 58, 62, 72–74] but also for most supposed plant mimics. Moreover, since the hymenopterans were already highly diversified at that time, a competition free space with similar morphologies would have helped Polygalaceae keel flowers to benefit this new area and radiate ([54, 75], Anonymous Reviewer 2, pers. comm.). For instance, for the orchid genus *Ophrys* L., it was suggested that diversification of the genus began after the well establishment of hymenopterans and because of the coevolution of plants and their pollinators, adaptive radiation has caused a species burst in the genus [75]. Furthermore, when two species share the same pollinator with the same flowering time and distribution, not only convergence of some floral traits (e.g., shape and color) to attract the same suit of available pollinators, but also divergence in others (i.e., different pollination niches) (e.g., odor) to prevent hybridization is common [75, 76]. Indeed, this floral adaptation under pollinator pressure (i.e., sharing the same suits of pollinators, 10) might have caused tribe Polygaleae to modulate its pollinator niche (Anonymous Reviewer 2, pers. comm.), and evolution of a similar floral bauplan with the Papilionoideae keel flowers. Therefore, in order to better understand keel flower evolution, it is necessary to conduct detailed comparative studies on keel flowers and their pollinators. For instance, while tripping mechanisms are reported for keel-flowered Polygalaceae [77], for other keel-flowered lineages among angiosperms the situation is unknown. Similarly, choice tests of keel flower pollinators, especially with naïve bees may reveal whether these pollinators move freely between different angiosperm keel flowers or not, and whether mimics (i.e., Polygalaceae keel flowers) receive more visits when they co-occur with the model (i.e., Fabaceae keel flowers), or mimics are more successful in terms of reproductive biology with the presence of legume keel flowers or not [40].

## Conclusions

To determine whether the first-evolving Polygalaceae keel flowers were functionally similar to existing Papilionoideae keel flowers and pollinated by similar pollinators, we carried out molecular dating, ancestral area and ancestral state analyses. The current study is the first to use ancestral reconstructions of traits associated with pollination to demonstrate that the multiple evolutionary origins of the keel flower pollinator syndrome in Fabales are consistent with, though do not prove, mimicry. Our results have shown that Polygaleae flowers might have benefited from existing Papilionoideae pollinators that were already visitors to Papilionoideae keel flowers. However, further research is needed to establish a better understanding of the context of the pollinators of keel flowers of different angiosperm clades. Whether other angiosperm clades that have keel flowers might have benefited from existing pollinators of keel-flowered Fabales, or vice-versa, could be addressed using the approaches we present here and a more inclusive phylogeny. Better understanding of the pollination biology of keeled and non-keeled genera of both Fabaceae and Polygalaceae would also be informative. In the field, research to better understand the pollination biology could include investigations to determine whether (1) there is any interspecific facilitation or competition (or both) between co-existing keel flowers [53]; (2) there are phenological differences or pollinator choice differences among co-existing keel flowers [53, 55, 78, 79]; (3) detailed measurements of floral parts (e.g., keel/flag length, colour, minor floral shape differences) are suggestive of shared pollination niche [75]; (4) there are shared main and secondary pollinators; (5) there are different body positions of the pollinators during pollen removal and pollination; (6) there is any evidence of adaptation of mechanical parts of the flowers [75]; (7) population sizes and plant densities of the keel flowers effect pollination success [75]; (8) rewards are [54] offered by sympatric keel-flowered species and (9) whether keel-flowered species share odours. On the other hand, future studies should also focus on the nectar-free Fabales keel flowers, particularly whether these species have more colorful and showy flowers, different odor emissions, high specialization rates, lower levels of pollination and/or pollination with mostly naïve bees, smaller geographical ranges and whether these taxa might have undergone a more rapid and extensive radiation compared to others, as in the case of orchids ([76]; Anonymous Reviewer 2, pers. comm.). Ultimately however, even if the pollination biology of extant species was suggestive of mimicry and a phylogenetic study supported it, an ancient mimicry scenario cannot be as robustly tested as a contemporary mimicry.

## Methods

### Taxon sampling and sequence data

Our phylogeny was reconstructed using published *matK*, *rbcL* and *trnL* plastid gene regions for 678 taxa, with 43 Fabidae outgroups. We reconstructed our phylogeny from the most widely available sequence data for all families of the Fabales. Whilst recent studies have employed considerable more sequence data (e.g., [80–82]), these data are not presently available across the Fabales. The monophylly of Fabales has been strongly supported; yet, both the fossil record and molecular studies (e.g., [12, 17, 48, 49]) suggest a rapid radiation for the order, which causes unstable phylogenetic relationships among the four families, namely, change in the topology and the root of the order by the choice of genes, outgroups and phylogenetic methods. Similarly, the evolutionary origin of the six subfamilies of Fabaceae has been reported as near simultaneous [81]. While the current study exceeds the taxon sampling of Bello et al. [12, 17], Aygoren Uluer et al. [49] and Koenen et al. [81, 82], it is the same as Aygoren Uluer et al. [48]; however, none of these studies were able to support a robust topology for the order. Furthermore, Koenen et al.’s [81] recently published phylogeny of the early evolving Leguminosae based on 1103 nuclear orthologues included only two Polygalaceae and one Surianaceae taxa, and therefore, it would not be possible to explore our molecular dating and ancestral reconstructions on their trees. Yet, it is encouraging that the previous attempts, particularly Koenen et al.’s [81] reconstructions in earliest evolving Papillionoideae are congruent to the current one. Furthermore, while we acknowledge that more data might indeed yield a higher resolution and support in the future; however, we believe that by using sets of Bayesian trees, we did not introduce spurious accuracy where data are not decisive.

The dataset contained the *matK*, *trnL* and *rbcL* plastid gene regions for 678 taxa (615 Fabaceae, 14 Polygalaceae, five Surianaceae and the sole genus of Quillajaceae, *Quillaja*). The GenBank numbers for these taxa are provided in (Appendix 1). Our sampling strategy was designed to include one species from each Fabales genera and Fabidae families as outgroups (43 outgroup taxa from Celastrales, Cucurbitales, Fagales, Malpighiales, Oxalidales, Rosales, Zygophyllales). Our sampling corresponds to 80% of Fabaceae genera (3% of species number) and 70% of Polygalaceae genera (1.4% of species number).

### Alignment, phylogenetic analyses

We assembled, trimmed, and aligned the sequences by using Geneious Pro 4.8.4 [83]. All indels were treated as missing data in all analyses.

The data matrix consisted of 3894 characters from 43 outgroup and 635 ingroup taxa, and 2445 (63%) characters were variable. Of these, 477 (12%) characters were parsimony uninformative, while 1968 (51%) characters were parsimony informative.

Maximum likelihood (ML) analyses were performed with RAxML [84], under a gamma model of heterogeneity with 1000 bootstrap replicates and with defined outgroup taxa and partitions for each data set and gene.

### Model choice and molecular dating analyses

For divergence time estimates we used BEAST v.1.8.0 [85]. The alignment was imported in BEAUti v.1.8.0 to [86] generate BEAST input files. BEAST was allowed to perform 2 × 10^7^.

MCMC generations, sampling every 1000th generation. We used a Yule process with a randomly generated starting tree and a lognormal relaxed model (uncorrelated) [87]. By using jModelTest2.1.10 [88, 89], for each of the genes, the most appropriate model was selected as GTR+G+I. Our study uses 30 fossil (24 ingroup and 6 outgroup) calibrations (Table 2). Other than the two relatively recent fossils, these fossil calibrations were taken from Lavin, Herendeen & Wojciechowski [90], Bruneau et al. [91] and Simon et al. [92]. Furthermore, six outgroup fossils were adopted for the first time in the context of a Fabales phylogeny.

**Table 2:** Fossils used to calibrate the Fabales tree

Name	Node constrained	Fossil organ(s)	Geographic location	Age (Ma)	References
A	Fabaceae stem node	Early fossil record of Fabaceae	Various locations	60–70	[90, 92]
C	*Cercis* stem node	*Cercis* leaves and fruits	Western North America	36	[93]
D	*Bauhinia* stem node	*Bauhinia* s.l. leaves	Tanzania	46	[91, 94, 92]
E	*Hymenaea* stem node	*Hymenaea* flower	Dominican Republic	24	[90–92, 92]
F	Mrca of *Prioria* and *Oxystigma*	*Prioria* flowers	Dominican Republic	24	[91, 96, 92]
F2	Mrca of clade of *Dimorphandra* group	*Protomimosoidea buchanensis* flowers	Tennessee, USA	55	[90–92, 90, 92]
G	*Daniellia* stem node	*Daniellia* wood	France	53	[91, 99, 92]
H	*Aphanocalyx* stem node	*Aphanocalyx l*eaves	Tanzania	46	[91, 100, 92]
I	*Crudia* stem node	*Crudia* fruits and leaflets	SE USA	45	[91, 101, 92]
I2	Stem node leading to *Styphnolobium* and *Cladrastis*	*Styphnolobium* and *Cladrastis* fruits and leaves	Tennessee, USA	40	[102, 90, 92]
J	Papilionoideae stem node	*Barnebyanthus buchananensis f*lowers	SE USA and Wyoming, USA	55	[90–92, 90, 92]
J2	Genistoid crown node	Leaves and pods similar to *Bowdichia* and *Diplotropis*	Western Wyoming, USA	56	[105, 90, 92]
K	*Swartzia* stem node	*Swartzia* fruits and leaflets	SE USA	45	[91, 102, 92]
K2	*Machaerium* stem node	Leaflets	Northern Mississippi, USA	40	[102, 90, 92]
L	*Arcoa* stem node	*Prosopis linearifolia* leaves	Florissant Locality, USA	34	[90–92, 92]
L2	Mrca of *Tipuana* and *Maraniona*	*Tipuana* fruits	Southern Ecuador	10	[107, 90, 92]
M	Mrca of *Acrocarpus*	*Acrocarpus* fruit	SE USA	45	[91, 92]
M2	*Robinia* stem node	*Robinia zirkelii* wood	North America and Europe	34	[108, 90, 92]
N	*Senna* stem node	*Senna* fruits	SE USA and Mexico	45	[91, 109, 102, 92]
O	*Caesalpinia* stem node	*Mezoneuron* fruits	SE and W USA	45	[90–92, 92]
Q	Mrca of Acacieae/Ingeae	Ingeae/Acacieae fossil pollen	Egypt	45	[91, 111, 92]
R	*Dinizia* stem node	*Eumimosoidea plumosa* flowers, leaves and fruits	SE USA	45	[91, 112, 101, 92]
X	*Calliandra* stem node	*Calliandra* pollen	Argentina	16	[113, 92]
Y	*Newtonia* stem node	*Newtonia* seeds	Ethiopia	21	[114]
124	Cucurbitaceae stem node	*Cucurbitospermum sheppeyense* seeds	London, UK	48.6	[115, 116]
117	Fagales stem node	*Archaefagacea futabensis* flowers and fruits	North-eastern Honshu, Japan	87.5	[116, 117]
121	Juglandaceae plus Myricaceae stem node	*Caryanthus* flowers and fruits	Georgia, USA	83.5	[116, 118]
123	Betulaceae stem node	*Bedellia pusilla* flowers	Georgia, USA	83.5	[116, 118]
113	Rhamnaceae stem node	*Coahuilanthus belindae* flowers	Coahuila, Mexico	70.6	[119, 116]
115	Ulmaceae stem node	*Ulmites* leaves	Northern Hemisphere sediments	55.8	[116, 120]

Sources for the calibration points are provided in the table. Outgroup fossils were adopted from Magallón et al. [116]

*Mrca* most recent common ancestor, *Ma* million years ago

The two new fossils used in this study are: (1) fossil leaves and fruits of *Cercis* from Oregon, USA with ~ 36 Ma age [93] (calibration point C). We did not perefer to use the 34 Ma old *Cercis* fossil [106] which was used by Lavin, Herendeen & Wojciechowski [90], Bruneau et al. [91] and Simon et al. [92], instead we used this recently described fossil in the current study because it represents the oldest fossil record of *Cercis* (Herendeen, pers. comm.). The 34 Ma old *Cercis* fossil was attributed to *C. herbmeyeri* Jia & Manchester, based on the “presence of indehiscent pods with a wing like flange along one margin” [106]. The fossil specimen was collected by S. R. Manchester and students from Teater Road, Crook County, Oregon in the 1980s, and was dated radiometrically to ~ 36 Ma with the help of an age for a nearby location, namely White Ash of Post tuff [106]. (2) fossil seeds of *Newtonia* from Ethiopia [114]. This earliest fossil record for the genus dates to 22–21 Ma, and used in the current study for the calibration point C (Herendeen, pers. comm.). A fossil of *Newtonia mushensis* Pan, Currano, Jacobs, Feseha, Tabor et Herendeen is the earliest and only definitive *Newtonia* fossil, was collected from The Mush Valley deposits, and dated by U-Pb radioisotope dating Method [114]. This new taxon is distinguished by seed size and seed characteristics (e.g., “flat, elongate tapering seeds bearing a membranous wing the degree of curvature near the funiculus attachment”) [114]. We also preferred to include Lavin, Herendeen & Wojciechowski’s [90] 60–70 Ma legume stem node constraint to the molecular clock rooting analysis (i.e., uniform prior distribution) for two reasons: (1) Lyson et al. [121] indicated that the Fabaceae oldest fossil record corresponds to 65.35 Ma, and this age is within the range we used to constrain the 60–70 Ma legume stem node, and (2) any convincing Fabaceae fossils prior to ca. 58 Ma are lacking [59, 90, 92, 101, 122]. To accommodate for gaps in the fossil record and uncertainity in fossil age estimates [105, 123], other than the 60–70 Ma legume stem node constraint [90], we used lognormal prior distribution with minimum age constraints. No fossils from Polygalaceae (e.g., [124–127]) and Surianaceae (e.g., [128, 129]) were used due to their unconfirmed status [12, 39, 90].

All BEAST analyses were implemented online via the CIPRES Portal [130]. Two independent runs were combined using LogCombiner v.1.8.0 [131]. Tracer v.1.6 [132] was used to check for proper mixing and convergence. TreeAnnotator v1.8.0 [133] was used to elect the maximum clade credibility trees. To annotate the tree, Interactive Tree of Life (iTOL) [134] was used.

### Ancestral area reconstruction

Eight biogeographic regions were defined according to Buerki et al. [47], with one addition. These areas were: A: Eurasia; B: Africa; C: Madagascar; D: Southeast Asia including Pacific Islands; E: Australia including New Guinea, New Caledonia and New Zealand; F: North America; G: South America including Central America and H: the Indian subcontinent. Here, area H includes India, Pakistan, Sri Lanka and Bangladesh, but not Nepal. The Indian subcontinent was treated as a separate area (H) in this study due to its recent (ca. 55 Ma) collision with the Eurasian plate [135]. The crown age of Fabales was estimated to be 84 Ma (maximum) by Bello et al. [12]. Therefore, taking the Indian subcontinent as a separate geographic area would be appropriate.

Geographic information for legume taxa was obtained from Legumes of the World [14]. For non-legume taxa, biogeographic information was obtained from published sources. Geography was scored at generic level for both legume and non-legume taxa, rather than for the species that were actually sampled. However, for the purpose of consistency, some clarifications are needed. First, the regions or countries covering more than one of the geographic regions were coded as: Mexico: F+G; Himalayas: A+H; Pakistan: A+H; pantropical, tropics or circumtropical: all areas from A to H; neotropical: F+G; paleotropical: A+B+C+D+E+H; subtropics: A+B+E+F+G; Southeast Asia: A+D; Asia: A+D+H. Second, when literature referred to a centre of diversity, this was taken as the distribution area. For instance, for a genus with a centre of diversity containing 30 spp. in North America, and one species with pantropical distribution, North America was accepted as the distribution area.

Biogeographical analyses were performed on our Bayesian tree with 678 taxa. The Lagrange (Dispersal-Extinction-Cladogenesis, DEC model; [136, 137]) option of RASP v.4.2 (Reconstruct Ancestral State in Phylogenies; [138]), was employed with default settings to calculate probabilities of the most likely ancestral areas for each clade (Additional file 3: S3), except the “maximum number of areas” option, which was set to 2 (the minimum), 4 and 6 to compare the results. A larger value (8, the maximum number of areas) was not tested; we specified the maximum number of areas as 4 since varying numbers of areas only slightly modified probabilities. The notable changes were for Fabaceae: the ancestral origin was 52% G or 49% BG if the maximum number of areas was set to 2, but 67–74% B+G or 33–26% G if the maximum number of areas was set to 4 and 6.

We applied a stratified biogeographical model by dividing our model into four time slices: before 80 Ma, between 80 and 65 Ma, between 65 and 30 Ma, and 30 Ma to the present day [47]. We also applied Buerki et al.’s [47] *Q* matrix in our ancestral area analysis, in which transition rates were dependent on the geographic location of areas.

### Ancestral trait reconstructions

Aygoren Uluer’s [70] review shows that the actual pollinators (rather than visitors) are known for only 33 keel flowered species of Fabales. The present study uses trait data from literature review of hundreds of published papers, and all available Floras score the traits that can be used to infer pollination syndrome. Many floral traits contribute to pollinator attraction. These include flower type, floral (corolla) symmetry, fusion of floral parts, flower size, length of nectar tube, inflorescence size, number of flowers in an inflorescence, inflorescence type, flower colour, floral reflectance, habit, height, height of flowers from ground, phenology, and floral scent [22, 28, 56, 139–145]. Having a bilateral symmetry, a pentamerous corolla with three different petal types, with the reproductive organs enclosed by keel petals and generally with connation of floral parts such as stamens and keel petals are also the essential characters of keel flowers [1–10]. In the current study, eleven morphological traits were selected as potentially the most important from the point of view of a pollinator (explained in detail below) and traced: floral type (keeled or not), presence or absence of a pentamerous corolla (petals+sepals in Polygalaceae), presence or absence of three distinct petal types (petals+sepals in Polygalaceae), presence or absence of enclosed reproductive organs, floral symmetry, androecium type, inflorescence type, inflorescence size, flower size, height and habit (Table 3).

**Table 3:** Explanation of eight pollination syndrome characters coded as A, B and/or C

Character	Character state			
Inflorescence type	A = Sequenced inflorescences (i.e., vertical inflorescences): raceme, panicle, spike, thyrse	B = Cluster type inflorescences (i.e., horizontal inflorescences): umbel, cyme, corymb, head, spike, fascicle		C = Solitary flowers
Habit type	A = Tall plants: tree, climber, liana, vine, scrambler	B = Medium plants: shrub, subshrub		C = Small plants: herb
Androecium type	A = Free stamens	B = Fused stamens		C = Polymorphic
Presence or absence of three types of petals (or petals+sepals in Polygalaceae),	A = YES	B = NO		C = The presence or absence of only two types of petals (or petals+sepals in Polygalaceae)
Presence or absence of a pentamerous corolla (pentamerous petals+sepals in Polygalaceae)	A = YES	B = NO		
Presence or absence of enclosed reproductive organs	A = YES	B = NO		
Presence or absence of a bilateral symmetry	A = Radial symmetry (including slightly bilateral symmetry)	B = Bilateral symmetry		
Keeled or not keeled	A = Keeled	B = Non-keeled		

1.a. The first character scored flowers as keeled or not keeled (Table 3, Additional file 4: S4). For this ancestral floral type analyses, we coded two states: A = keeled and B = non-keeled. In this case, Bello et al. [1] did not accept *Xanthophyllum* Roxb. flowers as keeled, but Van der Meijden [50] reported that some *Xanthophyllum* species may have keel flowers. On the other hand, Breteler and Smissaert-Houwing [146] reported that both *Carpolobia* G. Don and *Atroxima* Stapf have a keel petal which encloses the style and the stamen sheath similar to the Papilionoideae keel flowers. Unlike Van der Meijden [50], these authors avoided using the term papilionate flowers. Therefore, following Bello et al. [1], we did not accept *Carpolobia* and *Atroxima* as keel-flowered. In contrast, we coded *Xanthophyllum* as polymorphic for the character of being keel flowered or not.

The keel flower trait is known a priori to have more than one origin, and the purpose of our analyses are to highlight the recurring origin of the trait in a much more transparent and explicit way. We note however that non-keel flowers within Papilionoideae are not homologous, and referring to all of them as non-keeled may mislead analyses seeking to understand the transitions to keel morphologies [4]. With this in mind, we also divided the keel-flower trait into five further sub-traits which are: presence or absence of a pentamerous corolla (pentamerous petals+sepals in Polygalaceae), presence or absence of three types of petals (or petals+sepals in Polygalaceae), presence or absence of enclosed reproductive organs, presence or absence of a bilateral symmetry and androecium type [7, 9, 147] (Table 3, Additional files 5: S5, 6: S6, 7: S7, 8: S8, 9: S9).

1.b. For the presence or absence of a pentamerous corolla (pentamerous petals+sepals in Polygalaceae) analyses, we coded two states: A = YES and B = NO (Table 3).

1.c. For the presence or absence of three types of petals (or petals+sepals in Polygalaceae), analyses, we coded three states: A = YES, B = NO and C = the presence or absence of only two types of petals (or petals+sepals in Polygalaceae) (Table 3).

1.d. For the presence or absence of enclosed reproductive organs analyses, we coded two states: A = YES and B = NO (Table 3).

1.e. For the floral symmetry analyses, we coded two states: A = radial symmetry (including slightly bilateral symmetry) and B = bilateral symmetry (Table 3).

1.f. For the androecium type analyses, we coded three states: A = free stamens, B = fused stamens and C = polymorphic (Table 3). In this case, if the stamens are united at the base, we accepted this as free, due to a possible visual impact for pollinators. Although we code fusion of the androecium, we think that fusion of the petals is particularly common among later-diverging Papilionoideae tribes [6, 7, 9, 147]. Therefore, we did not include this character to our ancestral state analyses.

2. Inflorescence architecture is another important factor that affects pollinator visitation [56, 140, 148]. For example, two-dimensional inflorescences receive more hummingbird visits than three-dimensional ones [149]. Likewise, for vertical inflorescences, such as racemes, pollinators generally move from the bottom upwards (i.e., from oldest to youngest flowers) and starts to forage at the next inflorescence in this exact way [148, 150, 151]. However, it is not possible to find the same pattern on horizontal inflorescences such as umbels or heads. For these reasons, it is possible that for a pollinator, the visual impact of the inflorescence may be more important than the type of inflorescence, with convergent evolution on function between different inflorescence types. In other words, there might not be much visual difference between a panicle and a raceme in terms of what a bee sees. For these reasons, inflorescence morphology was coded as: A = Sequenced inflorescences (i.e., vertical inflorescences): raceme, panicle, spike and thyrse; B = Cluster type inflorescences (i.e., horizontal inflorescences): umbel, cyme, corymb, head, spike and fascicle; C = Solitary flowers (Table 3, Additional file 10: S10). It might have been informative to break this character down to overall shape of inflorescence, perhaps as width to length ratio, but this information was not available for all taxa.

3. Similarly, by effecting the foraging time of bees, not only the number of flowers in an inflorescence [152], but also the inflorescence size [140, 153] are other important criteria for pollinator attraction. While we could not obtain sufficient information about the number of flowers per inflorescence, we traced ancestral inflorescence sizes of first keel-flowered lineages of both Fabaceae and Polygalaceae. Both floral size and inflorescence size were scored in millimetres, and we considered both the smallest and the largest reported sizes for all taxa (Additional files 11: S11 and 12: S12, respectively).

4. Flower size is frequently reported to be an important part of pollinator attraction and therefore pollinator visitation [40, 145, 154–156]. The correlation between flower size and pollinator size (e.g., [157]), flower size and pollinator visitation rate (e.g., [158]), flower size and searching time (e.g., [145]) were shown by several studies; however, the results are almost always case dependent. Furthermore, while Fabaceae contains great diversity in flower size, from species with tiny flowers only a few millimetres in length (e.g. *Trifolium* L.) to giants (e.g., *Erythrina* L.), in Polygalaceae small flowers predominate. Therefore, we find it necessary to investigate ancestral floral sizes of first keel-flowered lineages of both Fabaceae and Polygalaceae.

5. Since pollinators tend to forage at constant heights to decrease flight distances [159–162], another important criterion for the study was height of plants and inflorescences from ground. However, since it is not always possible to find information about the inflorescence height from ground, approximations were made based on habit and height of plants, since both are frequently reported in the literature. We coded height of plants in centimetres, and we considered both the smallest and the largest reported sizes for all taxa (Additional files 13: S13 and 14: S14, respectively).

6. For the habit analyses, we coded three states: A = Tall plants: Tree, climber, liana, vine, scrambler; B = Medium plants: shrub, subshrub; C = Small plants: herb (Table 3, Additional file 1: S15).

Unfortunately, some traits could not be included in the current study due to the scarcity of information. These included UV reflectance (e.g., FReD: the floral reflectance database, [166]), presence or absence of pollen, presence or absence of secondary pollen presentation, presence or absence of nectar, length of nectar tube, height of flowers from ground and number of flowers in an inflorescence. Tracing others, such as phenology and floral colour was not possible due since we scored traits at generic level. Mapping global phenological data of a legume genus resulted a year-long flowering season for that taxon. Similarly, tracing the colour trait resulted as “all colours” for a genus, due to the occurrence of many differently coloured flowers of a large genus around the world. The 11 characters were scored for 635 taxa (excluding outgroups) in the four Fabales families. Data for these morphological traits were gathered from hundreds of appropriate, previously published sources including floras, articles and online sources (Additional file 1: S16). Our data are presented in the same linear order as the phylogenetic classification of Lewis [14], Gagnon et al. [163] and LPWG [15]. For all analyses, missing data were coded as "-".

In our analyses, we did not score geography and morphology at the species level, because our aim was to reflect the diversity within each genus, not each species. For example, while the flower size ranges from 3 mm to 2.5 cm in genus *Polygala* (e.g., *P*. *triflora* vs. *P*. *karensium*), in terms of pollination, scoring *Polygala* flowers as 3 mm or 25 mm would not be meaningful. The same logic applies here for at least floral type, habit, height, inflorescence size and inflorescence type analyses. Therefore, we think that scoring both the geography and morphology at the genus level is more appropriate in our situation.

Excluding outgroups which were scored as missing data, the amount of missing data for the flower type and ancestral area analyses was 0%, 0.5% for the presence or absence of three distinct petal types (petals+sepals in Polygalaceae) analysis, 2.8% for the presence or absence of enclosed reproductive organs analysis, 1.1% for the floral symmetry analysis, 8.7% for the androecium type analysis, 3.78% for the presence or absence of a pentamerous corolla (petals+sepals in Polygalaceae) analysis, 2.99% for the inflorescence type analysis, 15.9% for both the smallest and the largest flower size analyses, 16.38% for the smallest height analysis, 7.87% for the largest height analysis and 1.4% for the habit analysis. Unfortunately, the amount of missing data for both the smallest (46.6%) and the largest (42.2%) inflorescence size analyses was very high, due to the scarcity of information. For this reason, we could not estimate ancestral inflorescence sizes for most of the clades and ultimately we excluded ancestral inflorescence size analyses from our study.

To account for phylogenetic uncertainty, ancestral state reconstructions were performed on a sample of bootstrap trees with branch lengths. Since the phylogenetic relationships of the early-branching Papilionoideae are better resolved in our ML tree(s), we preferred to use the population of ML trees over our Bayesian tree. The program BayesTraits v2.0 [164, 165] was used for Bayesian estimation of ancestral states. For the “MultiState” model, MCMC analyses were run for 2 × 10^6^ generations, with default settings except the ratedev (rate deviation) and rjhp exp (RevJump) parameters and burn-in (the first 200,000 iterations). The “Continuous Random Walk” analyses were run with default settings, except the ratedev parameter. For the flower type analysis, we also conducted additional MCMC analyses for several Papilionoideae nodes in order to pinpoint the origin of keel flowers within the subfamily.

### Supplementary Information

**Additional file 1. S1.** Chronogram of Fabales.

**Additional file 2. S2.** Lagrange results of ancestral area reconstructions.

**Additional file 3. S3.** Input matrix for ancestral area analysis.

**Additional file 4. S4.** Input matrix for flower type analyses.

**Additional file 5. S5.** Input matrix for presence or absence of a pentamerous corolla analysis.

**Additional file 6. S6.** Input matrix for presence or absence of three types of petals analysis.

**Additional file 7. S7.** Input matrix for presence or absence of enclosed reproductive organs analysis.

**Additional file 8. S8.** Input matrix for presence or absence of a bilateral symmetry analysis.

**Additional file 9. S9.** Input matrix for androecium type analysis.

**Additional file 10. S10.** Input matrix for inflorescence type analysis.

**Additional file 11. S11.** Input matrix for the smallest flower size analysis.

**Additional file 12. S12.** Input matrix for the largest flower size analysis.

**Additional file 13. S13.** Input matrix for the smallest height analysis.

**Additional file 14. S14.** Input matrix for the largest height analysis.

**Additional file 15. S15.** Input matrix for habit analysis.

**Additional file 16. S16.** Sources used to construct ancestral state analyses of Fabales.

## Data Availability

All data generated or analysed during this study are included in the Additional files 1, 2, 3, 4, 5, 6, 7, 8, 9, 10, 11, 12, 13, 14, 15, 16.

## Appendix 1

Taxon sampling for the phylogenetic analyses of order Fabales based on the plastid *rbcL*, *matK* and *trnL*. A dash indicates the region was not sampled. GenBank accession numbers are presented in the following order: *rbcL*, *matK*, *trnL*.

**FABACEAE. Subfamily Duparquetioideae: *****Duparquetia*** —, EU361937.1, EU361800.1. **Subfamily Dialioideae: *****Poeppigia*** AY904370.1, AY386907.1, EU361829.1. ***Baudouinia*** —, EU361871.1, KJ620940.1. ***Eligmocarpus*** —, EU361939.1, EU361801.1. ***Mendoravia*** —, EU362001.1, EU361823.1. ***Distemonanthus*** —, EU361936.1, AF365084.1. ***Apuleia*** U74249.1, EU361858.1, EU361737.1. ***Storckiella*** AM234249.1, GU321970.1, AF365078.1. ***Labichea*** —, EU361989.1, AF365076.1. ***Petalostylis*** AF308719.1, AY386895.1, KJ620947.1. ***Koompassia*** —, EU361988.1, EU361816.1. ***Martiodendron*** —, EU361999.1, KJ620942.1. ***Zenia*** AF308722.1, EU362065.1, KJ620951.1. ***Dialium*** AM234245.1, EU361930.1, KJ620960.1. ***Dicorynia*** JQ626129.1, EU361931.1, KU356727.1. **Subfamily Cercidoideae: *****Cercis*** U74188.1, KT461986.1, FJ801163.1. ***Adenolobus*** AM234264.1, EU361845.1, FJ801158.1. ***Griffonia*** AM234265.1, JN881419.1, FJ801165.1. ***Brenierea*** AM234269.1, JN881409.1, —. ***Bauhinia*** KX119265.1, AY386893.1, MF135595.1. ***Gigasiphon*** JF738566.1, JN881416.1, FJ801108.1. ***Tylosema*** AJ584710.1, JN881457.1, FJ801124.1. ***Barklya*** —, JN881354.1, FJ801076.1. ***Lysiphyllum*** —, JN881430.1, FJ801152.1. ***Phanera*** —, JN881450.1, FJ801144.1. ***Lasiobema*** —, EU361873.1, FJ801138.1. ***Piliostigma*** JF265551.1, JN881454.1, —. **Subfamily Papilionoideae****: *****Bobgunnia*** AM234258.1, EU361885.1, AF365038.1. ***Swartzia*** AM234259.1, EU362053.1, KU728673.1. ***Candolleodendron*** —, JX295890.1, EF466264.1. ***Trischidium*** —, JX295868.1, JX275898.1. ***Cyathostegia*** —, AY553713.1, EF466267.1. ***Ateleia*** U74201.1, JX295883.1, EF466259.1. ***Amburana*** —, KX816341.1, EF466254.1. ***Mildbraediodendron*** —, —, AF309847.1. ***Cordyla*** U74204.1, JX295923.1, AF309848.1. ***Aldina*** U74252.1, KP177924.1, JX275891.1. ***Zollernia*** —, JX152595.1, JX275945.1. ***Holocalyx*** U74244.1, JX152593.1, JX187644.1. ***Lecointea*** AM234260.1, EU361990.1, JX187645.1. ***Harleyodendron*** —, JX152592.1, JX187643.1. ***Exostyles*** —, JX152590.1, JX187641.1. ***Baphiopsis*** —, JX295895.1, JX570586.1. ***Alexa*** JQ625719.1, JQ669613.1, KC178829.1. ***Castanospermum*** U74202.1, JX295891.1, AF311375.1. ***Angylocalyx*** U74200.1, JQ669611.1, AF311366.1. ***Xanthocercis*** U74189.1, JF270996.1, AF311365.1. ***Dussia*** U74206.1, JX295925.1, KC178835.1. ***Myrocarpus*** —, JF491270.1, JX275895.1. ***Myroxylon*** U74208.1, JX295912.1, JX275949.1. ***Myrospermum*** U74207.1, AY386959.1, AF309851.1. ***Cladrastis*** U74232.1, AY386861.1, AF311370.1. ***Styphnolobium*** KY872756.1, AY386962.1, KY872756.1. ***Calia*** —, AY386864.1, AF311374.1. ***Uribea*** —, AY553719.1, AF311000.1. ***Sweetia*** —, JX152620.1, JX187673.1. ***Luetzelburgia*** U74185.1, KX816379.1, JX187701.1. ***Ormosia*** JQ626235.1, KY079031.1, JX275921.1. ***Pericopsis*** U74210.1, —, —. ***Acosmium*** U74255.1, JX124417.1, JX124467.1. ***Bowdichia*** MG718274.1, JX124395.1, AF309486.1. ***Diplotropis*** JQ625878.1, JX124418.1, KC178836.1. ***Clathotropis*** —, KX584410.1, JX275957.1. ***Bolusanthus*** U74243.1, AF142685.1, AF310994.1. ***Platycelyphium*** —, —, AF309864.1. ***Dicraeopetalum*** —, GQ246142.1, JX275958.1. ***Cadia*** U74192.1, JX295932.1, AF309863.1. ***Ammodendron*** —, AY386957.1, —. ***Maackia*** AB127042.1, AY386944.1, MF444206.1. ***Sophora*** U74230.1, AF142693.1, JF338266.1. ***Salweenia*** U74251.1, —, —. ***Camoensia*** —, JX295919.1, KC178831.1. ***Dalhousiea*** —, —, AF310998.1. ***Airyantha*** —, JX295897.1, AF310997.1. ***Leucomphalos*** —, JX295864.1, KU324664.1. ***Baphia*** AM234261.1, EU361866.1, JX570590.1. ***Amphimas*** —, JX295894.1, KU324665.1. ***Panurea*** —, JX295947.1, JX275951.1. ***Spirotropis*** —, JX295950.1, KC178832.1. ***Taralea*** —, KX595211.1, JX275948.1. ***Pterodon*** —, AH009912.2, AF208895.1. ***Monopteryx*** —, JX295876.1, JX275906.1. ***Dipteryx*** JQ625725.1, JX295869.1, JX275899.1. ***Cyclolobium*** —, KJ028452.1, KX652197.1. ***Poecilanthe*** AB045818.1, KJ028459.1, KX652211.1. ***Harpalyce*** —, AF142689.1, —. ***Brongniartia*** U74253.1, GQ246147.1, —. ***Plagiocarpus*** —, GQ246160.1, —. ***Templetonia*** —, GQ246158.1, AF518122.1. ***Hovea*** Z95537.1, AY386889.1, AF518123.1. ***Lamprolobium*** —, GQ246159.1, —. ***Euchresta*** AB127040.1, —, AB127032.1. ***Pickeringia*** —, AY386863.1, —. ***Ammopiptanthus*** —, JQ820167.1, —. ***Anagyris*** Z70122.1, —, FJ499419.1. ***Cochliasanthus*** KF621120.1, —, KF621109.1. ***Piptanthus*** Z70123.1, AY386924.1, KP636629.1. ***Thermopsis*** JX848468.1, AY386866.1, HM590355.1. ***Baptisia*** Z70120.1, AY386900.1, FJ499421.1. ***Cyclopia*** AM261716.1, JX518243.1, —. ***Xiphotheca*** AM260746.1, —, —. ***Amphithalea*** AM235004.1, —, —. ***Stirtonanthus*** AM259368.1, —, —. ***Podalyria*** U74217.1, JX518039.1, —. ***Liparia*** AM259362.1, JX517632.1, —. ***Virgilia*** AM260739.1, JX517500.1, AF518125.1. ***Calpurnia*** U74239.1, AY386951.1, AF310993.1. ***Spartidium*** EU347931.1, —, —. ***Lebeckia*** EU347924.1, GQ246144.1, —. ***Wiborgia*** EU347975.1, —, —. ***Rafnia*** EU347896.1, JQ412281.1, —. ***Aspalathus*** EU348020.1, JQ412203.1, —. ***Lotononis*** Z95538.1, —, —. ***Bolusia*** EU347942.1, JQ040984.1, —. ***Crotalaria*** EU348034.1, AY386867.1, KP691152.1. ***Pearsonia*** EU347950.1, —, —. ***Rothia*** EU347953.1, —, —. ***Robynsiophyton*** EU347952.1, —, —. ***Melolobium*** Z95540.1, —, —. ***Dichilus*** EU347959.1, GQ246143.1, —. ***Polhillia*** EU347958.1, —, —. ***Argyrolobium*** Z95549.1, JQ412199.1, EU341594.1. ***Lupinus*** Z70070.1, AY386943.1, KX147711.1. ***Anarthrophyllum*** —, AY386923.1, AY618488.1. ***Adenocarpus*** Z95545.1, JQ858229.1, —. ***Cytisophyllum*** Z70090.1, —, AJ890966.1. ***Argyrocytisus*** Z70092.1, —, AJ890961.1. ***Petteria*** Z70091.1, —, AJ891026.1. ***Laburnum*** KM360837.1, HE967423.1, DQ417004.1. ***Cytisus*** HM849943.1, AY386902.1, MH000081.1. ***Calycotome*** —, —, JF338229.1. ***Echinospartum*** —, —, AF385415.1. ***Erinacea*** Z70105.1, —, AJ891029.1. ***Retama*** Z70117.1, —, AJ304872.1. ***Genista*** KM360800.1, AY386862.1, JF338287.1. ***Spartium*** HM850377.1, AY386901.1, JF338264.1. ***Stauracanthus*** —, —, AF385416.1. ***Ulex*** HM850431.1, JQ669586.1, AF385419.1. ***Apoplanesia*** —, AF270860.1, AF208898.1. ***Parryella*** —, AY391812.1, —. ***Amorpha*** U74212.1, KP126864.1, AF208899.1. ***Errazurizia*** —, AY391803.1, —. ***Eysenhardtia*** —, AY391807.1, —. ***Psorothamnus*** —, AY391818.1, —. ***Marina*** —, AY391811.1, —. ***Dalea*** —, AY391801.1, —. ***Vatairea*** AB045826.1, JX152606.1, JX187664.1. ***Vataireopsis*** JQ626110.1, AF142680.1, JX187670.1. ***Hymenolobium*** JQ625919.1, JX295903.1, JX275939.1. ***Andira*** U74199.1, JF501102.1, JX275929.1. ***Adesmia*** U74254.1, JN835371.1, —. ***Amicia*** —, AF203583.1, KF477933.1. ***Zornia*** U74235.1, KX595215.1, KF477982.1. ***Poiretia*** —, KX703011.1, —. ***Nissolia*** —, EU025907.1, AF208908.1. ***Chaetocalyx*** —, AF203585.1, KF477943.1. ***Riedeliella*** —, AH009910.1, MH603439.1. ***Discolobium*** —, AF270873.1, AF208964.1. ***Cranocarpus*** AB045796.1, AF270875.1, AF208951.1. ***Brya*** —, AF270876.1, AF208950.1. ***Platymiscium*** JQ626063.1, EU735957.1, EU736043.1. ***Platypodium*** GQ981836.1, AF270877.1, AF208961.1. ***Inocarpus*** JN083773.1, AF270878.1, AF208965.1. ***Maraniona*** KF436463.1, KF436439.1, —. ***Tipuana*** KF436476.1, AF270882.1, AF208956.1. ***Ramorinoa*** JN083776.1, AF270881.1, AF208957.1. ***Centrolobium*** JN083771.1, EU401414.1, EU401427.1. ***Paramachaerium*** KF436467.1, AF272062.1, AF208959.1. ***Etaballia*** KF436461.1, AH009902.1, AF208960.1. ***Pterocarpus*** MK026163.1, AF142691.1, AF208953.1. ***Cascaronia*** —, AF272072.1, AF208958.1. ***Geoffroea*** —, AF270880.1, AF208962.1. ***Fissicalyx*** —, AF272063.1, AF208938.1. ***Fiebrigiella*** —, AF203590.1, —. ***Chapmannia*** —, AF203593.1, AF208941.1. ***Stylosanthes*** JQ592043.1, NC_039161.1, KR737609.1. ***Arachis*** U74247.1, KX595195.1, JN617184.1. ***Grazielodendron*** JN083772.1, AF270862.1, AF208952.1. ***Dalbergia*** U74236.1, AF203582.1, KX268174.1. ***Machaerium*** U74248.1, KX898288.1, EF451120.1. ***Aeschynomene*** AF308701.1, AH009907.2, AF208927.1. ***Cyclocarpa*** —, AF272067.1, —. ***Soemmeringia*** —, AH009909.1, AF208937.1. ***Smithia*** —, AF272066.1, AF208933.1. ***Kotschya*** —, AF272065.1, KC560761.1. ***Humularia*** —, AF272069.1, AF208936.1. ***Bryaspis*** —, AF272068.1, AF208932.1. ***Geissaspis*** JQ933342.1, AF272064.1, AF208931.1. ***Pictetia*** —, AF203579.1, GQ889095.1. ***Diphysa*** JQ591721.1, AF203574.1, AF260645.1. ***Ormocarpum*** JX572809.1, AF203602.1, AF260646.1. ***Ormocarpopsis*** —, AF203567.1, AF208918.1. ***Peltiera*** —, GU951672.1, —. ***Weberbauerella*** —, AH009903.1, AF208909.1. ***Hypocalyptus*** AF308710.1, AY386886.1, AF518126.1. ***Gompholobium*** —, AY386891.1, AF518129.1. ***Sphaerolobium*** —, —, AF518136.1. ***Daviesia*** AF308708.1, AY386887.1, KY426137.1. ***Erichsenia*** —, —, AF518133.1. ***Viminaria*** —, —, AF518134.1. ***Isotropis*** AF308712.1, —, AY015083.1. ***Jacksonia*** —, AF298481.1, AF518147.1. ***Leptosema*** —, —, AF518148.1. ***Latrobea*** —, AF298484.1, AY883186.1. ***Euchilopsis*** —, —, AF113779.1. ***Phyllota*** —, AF298509.1, AF113785.1. ***Aotus*** —, AY386884.1, AY883181.1. ***Urodon*** —, —, AF113792.1. ***Stonesiella*** —, —, AF113791.1. ***Almaleea*** —, —, AF113775.1. ***Eutaxia*** —, —, AF113789.1. ***Dillwynia*** —, —, AF113778.1. ***Pultenaea*** —, —, AY883260.1. ***Mirbelia*** —, —, AY015087.1. ***Chorizema*** U74218.1, —, AF518154.1. ***Oxylobium*** —, —, AF113782.1. ***Podolobium*** —, —, AY015106.1. ***Callistachys*** —, AF298433.1, AY015072.1. ***Gastrolobium*** —, —, AY015082.1. ***Brachysema*** —, —, AY015071.1. ***Nemcia*** —, —, AY015099.1. ***Goodia*** U74258.1, —, KM876834.1. ***Platylobium*** —, —, KM876825.1. ***Muelleranthus*** —, —, KM876759.1. ***Ptychosema*** —, —, AF518165.1. ***Aenictophyton*** —, —, AF518144.1. ***Phylloxylon*** U74256.1, AY650280.1, AF274358.1. ***Cyamopsis*** —, AF142698.1, AF274359.1. ***Indigastrum*** —, —, AF274364.1. ***Microcharis*** —, AY650279.1, AF274363.1. ***Rhynchotropis*** —, —, AF274361.1. ***Vaughania*** —, —, KR738660.1. ***Indigofera*** U74214.1, AF142697.1, KX268190.1. ***Callerya*** AY308806.1, KP230744.1, AF124242.1. ***Endosamara*** AY308805.1, —, —. ***Afgekia*** GQ436345.1, —, —. ***Wisteria*** Z95544.1, AF142731.1, AF124239.1. ***Austrosteenisia*** U74242.1, AF142707.1, AF311381.1. ***Leptoderris*** AB045807.1, JX506609.1, —. ***Dalbergiella*** AF308724.1, AF142706.1, —. ***Aganope*** AF308702.1, JX506605.1, —. ***Ostryocarpus*** —, JX506599.1, —. ***Xeroderris*** AF308727.1, AF142708.1, —. ***Fordia*** AB045802.1, AF142718.1, —. ***Dewevrea*** AB045799.1, —, —. ***Craibia*** AB045795.1, JX517315.1, —. ***Disynstemon*** —, GU951670.1, —. ***Platycyamus*** AB045817.1, AF142709.1, KT696081.1. ***Kunstleria*** —, JX506598.1, —. ***Craspedolobium*** KF181485.1, JF953574.1, —. ***Philenoptera*** JF265547.1, JX506606.1, EU717357.1. ***Hesperothamnus*** AB045805.1, —, —. ***Piscidia*** AB045816.1, AF142710.1, AF311379.1. ***Deguelia*** AB045798.1, JX506607.1, —. ***Lonchocarpus*** AB045809.1, AF142717.1, AF311382.1. ***Muellera*** AB045813.1, —, —. ***Derris*** U74234.1, AF142715.1, AB817670.1. ***Paraderris*** —, JX506654.1, —. ***Millettia*** AF308714.1, AF142725.1, AF311377.1. ***Pongamiopsis*** AB045819.1, AF142711.1, KC479269.1. ***Chadsia*** AB045794.1, —, KC479260.1. ***Mundulea*** AB045814.1, AF142713.1, AY009136.1. ***Tephrosia*** Z95542.1, EU717429.1, KU750666.1. ***Apurimacia*** AF308703.1, FJ968527.1, —. ***Ptycholobium*** —, JQ669619.1, —. ***Abrus*** U74224.1, AF142705.1, EF543423.1. ***Dioclea*** AF308709.1, HQ707540.1, —. ***Canavalia*** AB045793.1, HQ707530.1, EU717354.1. ***Galactia*** AB045803.1, EU717428.1, KJ402390.1. ***Rhodopis*** AF308728.1, —, —. ***Ophrestia*** EU717289.1, AF142703.1, EU717359.1. ***Clitoria*** EU717286.1, EU717427.1, EU717355.1. ***Centrosema*** AF308706.1, JQ587552.1, —. ***Apios*** KJ773273.1, KF272942.1, EF543425.1. ***Shuteria*** AB045824.1, EU717423.1, LC228057.1. ***Mucuna*** MG946852.1, EU717422.1, KT696119.1. ***Kennedia*** EU717283.1, EU717424.1, KT696084.1. ***Hardenbergia*** EU717284.1, EU717425.1, EU717332.1. ***Vandasina*** —, —, EU717338.1. ***Spatholobus*** AB045825.1, EU106112.1, KF621116.1. ***Butea*** AB045789.1, JN008175.1, —. ***Adenodolichos*** AF308700.1, —, —. ***Paracalyx*** JQ933431.1, —, —. ***Bolusafra*** EU717272.1, EU717413.1, EU717309.1. ***Rhynchosia*** KF621126.1, JQ587827.1, MG709412.1. ***Eriosema*** KF621122.1, JQ587629.1, KF621111.1. ***Dunbaria*** JQ933314.1, —, —. ***Cajanus*** Z95535.1, EU717414.1, EF200131.1. ***Flemingia*** KF621123.1, KF621101.1, KF621112.1. ***Erythrina*** EU717270.1, EU717411.1, KX268185.1. ***Psophocarpus*** AB045820.1, JQ669575.1, EU717343.1. ***Otoptera*** —, JN008176.1, —. ***Decorsea*** —, AY582975.1, —. ***Strongylodon*** AF308729.1, —, —. ***Calopogonium*** AF308723.1, JQ669608.1, EU717318.1. ***Cologania*** EU717264.1, EU717405.1, EU717319.1. ***Pachyrhizus*** KJ468100.1, EU717401.1, EU717324.1. ***Neonotonia*** EU717261.1, EU717402.1, EU717323.1. ***Neorautanenia*** AF308715.1, JN008178.1, —. ***Dumasia*** EU717265.1, EU717406.1, EU717320.1. ***Pueraria*** AB045822.1, AY582972.1, LC315108.1. ***Pseudeminia*** AF181936.1, —, —. ***Pseudovigna*** EU717262.1, EU717403.1, EU717325.1. ***Amphicarpaea (Amphicarpa)*** AF181930.1, EU717399.1, LC315110.1. ***Teramnus*** EU717258.1, EU717400.1, LC315106.1. ***Glycine*** Z95552.1, AF142700.1, JN617170.1. ***Phylacium*** AB045815.1, —, —. ***Wajira*** —, AY583011.1, —. ***Sphenostylis*** —, AY582978.1, —. ***Nesphostylis*** —, AY582979.1, —. ***Alistilus*** —, JN008191.1, —. ***Dolichos*** AF413209.1, JN008183.1, —. ***Macrotyloma*** EU717269.1, AY589507.1, EU717341.1. ***Dipogon*** AB045800.1, AY582988.1, —. ***Lablab*** EU717267.1, EU717408.1, EU717339.1. ***Spathionema*** —, AY582990.1, —. ***Vatovaea*** —, JN008194.1, —. ***Physostigma*** —, AY582998.1, —. ***Vigna*** Z95543.1, AY582999.1, —. ***Oxyrhynchus*** AF308717.1, AY509935.1, —. ***Phaseolus*** KF022496.1, DQ450863.1, —. ***Ramirezella*** —, AY509936.1, EU717344.1. ***Strophostyles*** —, DQ443469.1, EU717345.1. ***Dolichopsis*** —, AY509943.1, —. ***Macroptilium*** EU717268.1, EU717409.1, EU717340.1. ***Mysanthus*** —, AY509941.1, —. ***Campylotropis*** EU717277.1, EU717418.1, JN402864.1. ***Kummerowia*** EU717276.1, EU717417.1, JN402866.1. ***Lespedeza*** KJ773624.1, EU717419.1, AB538914.1. ***Dendrolobium*** —, —, AB538884.1. ***Phyllodium*** —, HM049524.1, —. ***Tadehagi*** KX527094.1, KF621106.1, KF621117.1. ***Desmodium*** EU717280.1, EU717421.1, KM098861.1. ***Codariocalyx*** KX527051.1, KF621099.1, KF621110.1. ***Hylodesmum*** KF621124.1, KF621102.1, KM098857.1. ***Pseudarthria*** KY702624.1, JF270902.1, —. ***Uraria*** JQ933516.1, JN407137.2, KF621118.1. ***Christia*** KX527413.1, KF621098.1, KF621108.1. ***Alysicarpus*** JN628036.1, JQ587509.1, —. ***Otholobium*** U74219.1, JN008180.1, EU717351.1. ***Psoralea*** AM235013.1, JN008182.1, EU717352.1. ***Orbexilum*** —, EF549998.1, EF543345.1. ***Hoita*** —, EF549962.1, EF543367.1. ***Rupertia*** —, EF549999.1, EF543415.1. ***Psoralidium*** —, EF549941.1, EU717353.1. ***Pediomelum*** JX848465.1, EF549988.1, HM590336.1. ***Bituminaria*** U74221.1, JF501107.1, EU717349.1. ***Cullen*** EU717254.1, EF550002.1, EU717350.1. ***Sesbania*** Z95541.1, JX453721.1, —. ***Hippocrepis*** KF602185.1, JQ619986.1, KY697425.1. ***Securigera*** KM360978.1, AF543846.1, —. ***Coronilla*** U74222.1, JQ619970.1, HQ323870.1. ***Anthyllis*** KF602115.1, AF543845.1, KY697485.1. ***Ornithopus*** HM850217.1, AF142727.1, —. ***Lotus*** MG249841.1, AF142729.1, MF314953.1. ***Dorycnium*** FR865124.1, JQ619969.1, MF314954.1. ***Hammatolobium*** —, JQ619984.1, —. ***Hebestigma*** —, AF543850.1, AF400134.1. ***Lennea*** JQ591832.1, AF543851.1, AF400135.1. ***Gliricidia*** KX119294.1, AF547197.1, AF400138.1. ***Poitea*** —, AF547198.1, AF400141.1. ***Olneya*** —, AF543857.1, AF529393.1. ***Robinia*** U74220.1, AF142728.1, AF529391.1. ***Coursetia*** JQ591655.1, AF155814.1, AF529405.1. ***Peteria*** —, AF547190.1, AF529395.1. ***Genistidium*** —, AF543858.1, AF529394.1. ***Sphinctospermum*** —, AF547191.1, AF529392.1. ***Glycyrrhiza*** AB126685.1, AY386883.1, AF124238.1. ***Chesneya*** JQ933263.1, JQ669609.1, AB287413.1. ***Gueldenstaedtia*** KX021596.1, KX021479.1, KX021666.1. ***Tibetia*** KX021599.1, JQ619951.1, —. ***Erophaca*** —, JQ619937.1, —. ***Oxytropis*** HM142226.1, AY386915.1, LT994895.1. ***Biserrula*** —, AY920448.1, AF126995.1. ***Astragalus*** EF685984.1, AF142736.1, AF127001.1. ***Barnebyella*** —, JQ669593.1, —. ***Colutea*** JQ933276.1, AY386874.1, AF126993.1. ***Oreophysa*** —, —, AB287415.1. ***Smirnowia*** —, JQ669579.1, —. ***Eremosparton*** —, JQ619964.1, —. ***Sphaerophysa*** —, JQ669581.1, AF126996.1. ***Lessertia*** MF286764.1, AY920453.1, AF126997.1. ***Sutherlandia*** JQ025097.1, AY386913.1, AF126994.1. ***Swainsona*** —, AF142735.1, AF126999.1. ***Montigena*** JQ933414.1, —, —. ***Clianthus*** JQ933270.1, AY386914.1, AF126998.1. ***Carmichaelia*** AF308705.1, AY386873.1, MF597719.1. ***Galega*** —, JQ669610.1, AB854528.1. ***Calophaca*** KX942277.1, JF501109.1, AF124230.1. ***Caragana*** KX942272.1, AF142737.1, LC309038.1. ***Halimodendron*** FJ537237.1, JQ619947.1, AB854534.1. ***Alhagi*** —, AY386880.1, AB854520.1. ***Eversmannia*** —, AB854573.1, AB854527.1. ***Hedysarum*** JX848461.1, JQ669599.1, KY366138.1. ***Corethrodendron*** —, AB854567.1, AB854522.1. ***Sulla*** KC700648.1, —, —. ***Taverniera*** —, JQ669585.1, KY366151.1. ***Onobrychis*** KX942280.1, AY386879.1, KY697532.1. ***Sartoria*** —, AB854583.1, AB854535.1. ***Ebenus*** —, JQ619960.1, AB854525.1. ***Cicer*** AF308707.1, AY386897.1, JN617169.1. ***Parochetus*** JQ933432.1, JQ619993.1, DQ311716.1. ***Trifolium*** HM850420.1, AF522131.1, KX668031.1. ***Ononis*** KF602196.1, AF522114.2, GQ488607.1. ***Melilotus*** KP126850.1, AF522110.2, KX667997.1. ***Trigonella*** MG946901.1, AF522151.2, JX274159.1. ***Medicago*** Z70173.1, AF522108.2, —. ***Vicia*** JN661200.1, AY386899.1, JN617168.1. ***Lens*** KJ850239.1, AF522089.1, JN617171.1. ***Lathyrus*** MG946891.1, AF522084.1, LC311143.1. ***Pisum*** MG917089.1, JX505829.1, LC311179.1. ***Vavilovia*** JX505491.1, JX505832.1, KT757953.1. **Subfamily Caesalpinioideae: *****Chamaecrista*** AM234248.1, EU361914.1, KR737640.1. ***Senna*** U74250.1, EU362042.1, KC479270.1. ***Cassia*** AM234244.1, JQ619983.1, AF365092.1. ***Gymnocladus*** AY904373.1, EU361966.1, EU361814.1. ***Gleditsia*** AY904374.1, AY386930.2, EU361812.1. ***Umtiza*** AM234237.1, GU321973.1, AF365126.1. ***Tetrapterocarpon*** AY904372.1, JX099333.1, AY899684.1. ***Arcoa*** —, AY386933.1, AY232787.1. ***Acrocarpus*** EU361843.1, AY904371.1, AF365098.1. ***Ceratonia*** U74203.1, EU361911.1, AY232782.1. ***Pterogyne*** AY904377.1, EU362031.1, AF365074.1. ***Haematoxylum***** (*****Haematoxylon)*** AY904383.1, AY386905.1, AY899696.1. ***Cordeauxia*** AY904378.1, AY386918.2, EU361787.1. ***Stuhlmannia*** AY904395.1, JX099335.1, EU361839.1. ***Mezoneuron*** —, EU361903.1, KX373107.1. ***Pterolobium*** —, EU362032.1, AF365073.1. ***Tara*** —, —, HQ011837.1. ***Coulteria*** KU176172.1, JQ587526.1, —. ***Caesalpinia*** U74190.1, EU361906.1, KX373109.1. ***Pomaria*** —, EU362029.1, EU361830.1. ***Erythrostemon*** JN796934.1, JX099328.1, JX219458.1. ***Poincianella*** JX856660.1, EU361904.1, —. ***Cenostigma*** —, —, JX073262.1. ***Guilandina*** —, EU361900.1, KX373104.1. ***Libidibia*** —, EU361901.1, KX373119.1. ***Stahlia*** —, EU362050.1, EU361838.1. ***Hoffmannseggia*** AY308531.1, JQ619977.1, JX219459.1. ***Stenodrepanum*** —, JX219467.1, JX219462.1. ***Zuccagnia*** AY308547.1, JX219468.1, EU361842.1. ***Lophocarpinia*** —, JX219466.1, JX219460.1. ***Balsamocarpon*** AY308524.1, EU361864.1, JX219457.1. ***Moullava*** —, JX099331.1, JX073267.1. ***Batesia*** AY904375.1, EU361869.1, —. ***Recordoxylon*** JQ626133.1, —, AY899699.1. ***Melanoxylon*** AY904388.1, EU362000.1, EU361822.1. ***Moldenhawera*** AY904390.1, EU362004.1, EU361824.1. ***Tachigali***** (*****Tachigalia*****)** JQ625892.1, EU362040.1, KR872696.1. ***Arapatiella*** AY904376.1, EU361859.1, EU361738.1. ***Jacqueshuberia*** AY904392.1, EU361984.1, EU361815.1. ***Schizolobium*** AY904398.1, GQ167770.1, AY899711.1. ***Bussea*** AY904396.1, EU361896.2, AY899708.1. ***Peltophorum*** AY904401.1, KX538533.1, EU361828.1. ***Parkinsonia*** AY904403.1, EU362019.1, EF101295.1. ***Conzattia*** AY904416.1, AY386918.2, EU361786.1. ***Delonix*** AY904421.1, EU361928.1, KY040047.1. ***Colvillea*** AY904425.1, EU361916.1, AY899739.1. ***Lemuropisum*** AY904426.1, EU361991.1, AF430778.1. ***Pachyelasma*** —, EU362013.1, AF365105.1. ***Erythrophleum*** U74205.1, EU361948.1, JX840218.1. ***Dimorphandra*** —, EU361934.1, AF365099.1. ***Mora*** —, EU362005.1, —. ***Burkea*** JX572357.1, EU361895.1, EU361755.1. ***Stachyothyrsus*** —, JX099332.1, JX073268.1. ***Sympetalandra*** —, —, EU361840.1. ***Campsiandra*** —, EU361908.1, EU361780.1. ***Chidlowia*** —, JX099329.1, JX073263.1. ***Diptychandra*** —, EU361935.1, AF309478.1. ***Vouacapoua*** —, EU362063.1, AF365110.1. ***Dinizia*** —, JX295860.1, EU361798.1. ***Pentaclethra*** AM234250.1, AF521853.1, AF278485.1. ***Adenanthera*** —, AF521808.1, AF278486.1. ***Tetrapleura*** —, AF521865.1, AY125852.1. ***Amblygonocarpus*** JX572301.1, AF521812.1, AF278487.1. ***Pseudoprosopis*** —, AF521861.1, AY125851.1. ***Calpocalyx*** AM234257.1, EU361907.1, AF278483.1. ***Xylia*** JF265660.1, AF521866.1, AY125849.1. ***Piptadeniastrum*** —, AF521857.1, AF278488.1. ***Entada*** JQ025045.1, EU328448.1, EU328504.1. ***Elephantorrhiza*** JF265409.1, AF521828.1, AF278484.1. ***Plathymenia*** —, AF521858.1, AF278509.1. ***Indopiptadenia*** JQ388196.1, —, —. ***Newtonia*** KC628005.1, AF521848.1, —. ***Fillaeopsis*** —, AF521833.1, AY125847.1. ***Cylicodiscus*** —, AF521819.1, AY125845.1. ***Prosopis*** KF471677.1, AY574098.1, MG709383.1. ***Xerocladia*** —, EU000438.1, EU004653.1. ***Prosopidastrum*** —, EF165252.1, AY944543.1. ***Piptadeniopsis*** —, AY944559.1, AY944542.1. ***Neptunia*** KX119312.1, AF523090.1, AF278495.1. ***Leucaena*** —, AY574102.1, EU439990.1. ***Schleinitzia*** —, AF521862.1, AF278491.1. ***Desmanthus*** —, AF521820.1, EU440011.1. ***Kanaloa*** —, AF521839.1, AF278489.1. ***Calliandropsis*** —, AF521816.1, AF278520.1. ***Dichrostachys*** KX119290.1, JQ024956.1, KX268182.1. ***Alantsilodendron*** —, AF521811.1, AY125844.1. ***Parkia*** AM234251.1, EU362018.1, AF278499.1. ***Anadenanthera*** MH560445.1, EU812064.1, AF278486.1. ***Pseudopiptadenia*** JQ625948.1, DQ790637.1, FJ039253.1. ***Piptadenia*** JQ592111.1, DQ790616.1, DQ784675.1. ***Parapiptadenia*** —, DQ790610.1, DQ784653.1. ***Microlobius*** —, AF521842.1, AF522960.1. ***Stryphnodendron*** JQ626052.1, DQ790643.1, DQ784680.1. ***Adenopodia*** JF265272.1, JF270628.1, —. ***Mimosa*** JQ591939.1, AY944555.1, —. ***Mimozyganthus*** —, AY944557.1, AY944540.1. ***Acacia*** KX397681.1, AF274131.1, GQ872270.1. ***Faidherbia*** JF265429.1, HM020737.1, KY100268.1. ***Zapoteca*** JQ592091.1, EU362064.1, JX870899.1. ***Calliandra*** JQ591619.1, JQ587539.1, JX870877.1. ***Viguieranthus*** —, —, JX870892.1. ***Macrosamanea*** —, —, JX870883.1. ***Cojoba*** KJ082229.1, AY944554.1, AY944538.1. ***Inga*** FJ173744.1, EU361980.1, JX870880.1. ***Cedrelinga*** AM234256.1, AF521818.1, JX870873.1. ***Zygia*** JQ625977.1, JQ626423.1, JX870900.1. ***Archidendron*** AM234253.1, EU361860.1, KX852438.1. ***Paraserianthes*** HM850233.1, EU812040.1, EU440016.1. ***Pararchidendron*** —, AF274127.1, EU439985.1. ***Hydrochorea*** —, —, JX870879.1. ***Abarema*** JQ626162.1, GQ981925.1, JX870787.1. ***Blanchetiodendron*** —, —, JX870790.1. ***Leucochloron*** —, —, JX870882.1. ***Chloroleucon*** —, JQ587561.1, AF278517.1. ***Cathormion*** —, AF274122.1, AF522949.1. ***Thailentadopsis*** —, —, JX870889.1. ***Sphinga*** —, —, JX870887.1. ***Havardia*** JQ591981.1, AF274125.1, KF933280.1. ***Ebenopsis*** —, AY125853.1, KF921772.1. ***Pithecellobium*** KX119318.1, HM020740.1, JX870884.1. ***Pseudosamanea*** JQ591566.1, AF523079.1, JX870885.1. ***Samanea*** MH560455.1, AF523073.1, JX870886.1. ***Albizia*** KX119255.1, EU812047.1, JX870788.1. ***Enterolobium*** MG718296.1, AF523096.1, AF522953.1. ***Lysiloma*** JQ591891.1, AF274126.1, KF933281.1. **Subfamily Detarioideae: *****Neoapaloxylon*** —, —, KC479267.1. ***Schotia*** AM235016.1, EU362037.1, AF365124.1. ***Barnebydendron*** —, EU361868.1, AF365209.1. ***Goniorrhachis*** AM234232.1, EU361959.1, AF365185.1. ***Brandzeia*** —, EU361870.1, AY187226.1. ***Oxystigma*** —, —, AY958477.1. ***Kingiodendron*** JF739130.1, EU361987.1, AF365169.1. ***Gossweilerodendron*** —, EU361960.1, AF365166.1. ***Prioria*** —, EU362030.1, AF365171.1. ***Colophospermum*** JF265343.1, AY386894.1, AF365165.1. ***Hardwickia*** —, EU361967.1, AY187227.1. ***Daniellia*** —, EU361927.1, KX268175.1. ***Eurypetalum*** —, KX162081.1, AF365137.1. ***Eperua*** JQ626198.1, EU361945.1, FJ039225.1. ***Augouardia*** —, EU361862.1, AF365164.1. ***Stemonocoleus*** —, EU362051.1, AF365178.1. ***Peltogyne*** AF308718.1, EU362021.1, AY958483.1. ***Hymenaea*** JQ625969.1, EU361972.1, FJ009872.1. ***Guibourtia*** JX572650.1, EU361962.1, GQ889071.1. ***Hylodendron*** —, EU361971.1, AF365186.1. ***Gilletiodendron*** —, EU361957.1, AF365184.1. ***Baikiaea*** JX572322.1, EU361863.1, AY958459.1. ***Tessmannia*** —, EU362059.1, AF365192.1. ***Sindora*** —, EU362048.1, AY958486.1. ***Sindoropsis*** —, EU362049.1, AF365189.1. ***Copaifera*** —, EU361919.1, AF365181.1. ***Detarium*** AM234239.1, EU361929.1, AF365183.1. ***Endertia*** —, EU361943.1, AF365136.1. ***Lysidice*** —, EU361995.1, AF365152.1. ***Saraca*** AM234238.1, EU362035.1, AF365157.1. ***Talbotiella*** KC628128.1, EU362055.1, AF365159.1. ***Scorodophloeus*** —, EU362041.1, KF294049.1. ***Crudia*** AM234230.1, EU361922.1, AF365172.1. ***Lebruniodendron*** —, KF294060.1, EU361817.1. ***Plagiosiphon*** —, EU362025.1, KJ777465.1. ***Micklethwaitia*** —, KF294062.1, KF294045.1. ***Maniltoa*** FJ976148.1, EU361998.1, AF365121.1. ***Cynometra*** AM234231.1, EU361925.1, KF294041.1. ***Tamarindus*** AB378731.1, EU362056.1, KJ468103.1. ***Intsia*** KF496786.1, EU361981.1, AF365149.1. ***Afzelia*** MF437031.1, EU361848.1, AF365130.1. ***Brodriguesia*** —, EU361890.1, EU361750.1. ***Loesenera*** —, EU361994.1, AF233472.1. ***Neochevalierodendron*** —, EU362006.1, AF365151.1. ***Normandiodendron*** —, EU362007.1, AF233457.1. ***Zenkerella*** —, EU362066.1, AF365127.1. ***Humboldtia*** JX163311.1, EU361970.1, AF365214.1. ***Hymenostegia*** KC685085.1, KF294458.1, AF233470.1. ***Leonardoxa*** —, EU361992.1, AF233463.1. ***Amherstia*** AM234234.1, AF542601.1, AF365210.1. ***Ecuadendron*** —, EU361938.1, AF365207.1. ***Paloue***** (*****Palovea*****)** —, EU362016.1, FJ817583.1. ***Paloveopsis*** —, —, FJ817571.1. ***Heterostemon*** —, EU361968.1, FJ817569.1. ***Elizabetha*** —, EU361942.1, FJ817561.1. ***Brownea*** U74186.1, KF794162.1, EU361753.1. ***Browneopsis*** AM234233.1, EU361894.1, AF365199.1. ***Macrolobium*** JQ625745.1, MF946643.1, FJ817552.1. ***Paramacrolobium*** —, EU362017.1, AF365242.1. ***Cryptosepalum*** —, EU361923.1, AF365258.1. ***Dicymbe*** —, EU361932.1, JN168671.1. ***Polystemonanthus*** —, EU362028.1, AF365226.1. ***Englerodendron*** —, EU361944.1, AF365218.1. ***Anthonotha*** KC628021.1, KX161940.1, AF365233.1. ***Berlinia*** KC628285.1, EU361881.1, HM059918.1. ***Librevillea*** —, EU361993.1, KJ777457.1. ***Didelotia*** —, EU361933.1, KJ777380.1. ***Gilbertiodendron*** KC628266.1, —, KJ777451.1. ***Gilbertiodendron*** —, EU362020.1, AF365243.1. ***Isoberlinia*** AM234240.1, EU361983.1, AF365221.1. ***Oddoniodendron*** —, EU362009.1, AF365225.1. ***Microberlinia*** —, EU362003.1, AF365223.1. ***Julbernardia*** JX572701.1, EU361986.1, AF365266.1. ***Brachystegia*** KU568078.1, EU361886.1, AF365254.1. ***Tetraberlinia*** —, KX162318.1, AF365230.1. ***Bikinia*** —, EU361884.1, AY116897.1. ***Icuria*** —, EU361979.1, AF365232.1. ***Aphanocalyx*** AM234241.1, EU361855.1, AF365248.1. **POLYGALACEAE. Tribe Polygaleae: *****Bredemeyera*** EU644699.1, EU596520.1, GQ889062.1. ***Comesperma*** AM234179.1, EU596516.1, GQ889068.1. ***Monnina*** AM234184.1, EU604047.1, AM234275.1. ***Muraltia*** AJ829698.1, AM889730.1, GQ889090.1. ***Polygala*** EU644684.1, EU596518.1, GQ889213.1. ***Salomonia*** —, —, GQ889225.1. ***Securidaca*** EU644681.1, EU604029.1, GQ889230.1. **Tribe Moutabeae: *****Barnhartia*** AM234168.1, —, —. ***Diclidanthera*** —, —, AF366955.1. ***Eriandra*** AM234170.1, EU604051.1, GQ889070.1. ***Moutabea*** AM234169.1, JQ626362.1, AF366966.1. **Tribe Carpolobieae: *****Atroxima*** AM234175.1, EU604049.1, GQ889057.1. ***Carpolobia*** AM234176.1, EU604053.1, GQ889064.1. **Tribe Xanthophylleae: *****Xanthophyllum*** AM234229.1, JN564163.1, AF367004.1. **SURIANACEAE. *****Cadellia*** L29491.1, EU604056.1, AM234304.1. ***Guilfoylia*** L29494.1, EU604031.1, AF367010.1. ***Recchia*** AM234270.1, EU604045.1, AF367009.1. ***Stylobasium*** U06828.1, EU604032.1, —. ***Suriana*** U07680.1, AY386950.1, AM234306.1. **Q UILLAJACEAE. *****Quillaja*** U06822.1, AY386843.1, AF367008.1. **OUTGROPUS. Zygophyllales: *****Bulnesia*** EU002275.1, EU002172.1, AJ387947.1. **Celastrales: *****Celastrus*** KF022456.1, EU328939.1, EU328814.1. **Oxalidales: *****Oxalis*** JN587327.1, AF542605.1, JN620140.1. **Malpighiales: *****Viola*** JQ950611.1, JX661966.1, KU558527.1. **Rosales: *****Prunus*** AF329005.1, AF288116.1, MG773118.1. ***Barbeya*** AJ225788.1, JF317418.1, AJ225795.1. ***Rhamnus*** L13189.2, AF288121.1, KY193773.1. ***Spyridium*** AJ390058.1, AF049849.1, AY998780.1. ***Ulmus*** KM361026.1, AY257536.1, MG773098.1. ***Morus*** D86319.1, AY257531.1, KT207493.1. ***Ficus*** JQ773660.1, AY257530.1, EU191024.1. ***Cannabis*** AF500344.1, AF345317.1, KF250352.1. ***Humulus*** KM360825.1, AF345318.1, KY313870.1. ***Zelkova*** MF706362.1, AB572497.1, MF674002.1. ***Fragaria*** HM850009.1, AF288102.1, KU600394.1. ***Elaeagnus*** JX848456.1, AY257529.1, HM590275.1. ***Hippophae*** JF317488.1, JF954040.1, KU304418.1. ***Urtica*** AF500361.1, EU002192.1, MG773116.1. ***Urera*** AF500360.1, KF138066.1, MH358317.1. ***Dirachma*** JF317482.1, JF317423.1, AJ225796.1. **Fagales: *****Platycarya*** AY263933.1, AF118040.1, AY147078.1. ***Nothofagus*** L13357.2, U92860.1, AY745879.1. ***Gymnostoma*** AY033870.1, AY191695.1, —. ***Casuarina*** AY033854.1, U92858.1, AB817433.1. ***Juglans*** AY263932.1, MF167461.1, MG773097.1. ***Morella*** DQ310502.1, AY491657.1, MF503613.1. ***Myrica*** KM360891.1, AY191715.1, GQ245143.1. ***Betula*** L01889.2, AY372027.1, MG773096.1. ***Alnus*** NC_039930.1, AB038176.1, MF136516.1. ***Ticodendron*** AF061197.1, U92855.1, AY147073.1. ***Quercus*** MF044887.1, AB727873.1, MG773106.1. ***Castanea*** AF500363.1, EF057123.1, KF718309.1. **Cucurbitales: *****Anisophyllea*** AY973487.1, AY935923.1, AY968560.1. ***Combretocarpus*** AF127698.1, AY968447.1, AY968561.1. ***Bolbostemma*** DQ501255.1, DQ469139.1, DQ501264.1. ***Begonia*** U59814.1, GU397115.1, AY238597.1. ***Hillebrandia*** U59822.1, GU397085.1, AY968564.1. ***Datisca*** MH900512.1, AY968449.1, AY238601.1. ***Octomeles*** L21942.1, AY968455.1, AY968574.1. ***Tetrameles*** L21943.1, AY968458.1, AY091831.1. ***Corynocarpus*** AF148994.1, AY968448.1, HQ207704.1. ***Coriaria*** KF022461.1, AB016460.1, AY091825.1. ***Cucumis*** L21937.1, DQ785841.1, HM597059.1.

## Appendix 2

The Papilionoideae clades which evolved between ~ 49 and 45 Ma and their descendants.

**Clade 1**: *Candolleodendron, Swartzia*; **Clade 2**: *Pericopsis, Camoensia, Diplotropis, Bowdichia*; **Clade 3**: *Amicia, Zornia, Poiretia, Chaetocalyx, Nissolia, Adesmia*; **Clade 4**: *Kotschya*, *Humularia*, *Smithia*, *Cyclocarpa*, *Soemmeringia*, *Bryaspis*, *Aeschynomene*, *Ormocarpopsis*, *Peltiera*, *Ormocarpum*, *Pictetia*, *Diphysa*, *Dalbergia*, *Machaerium*, *Weberbauerella*; **Clade 5**: *Kotschya, Humularia, Smithia, Cyclocarpa, Soemmeringia, Bryaspis, Aeschynomene, Ormocarpopsis, Peltiera, Ormocarpum, Pictetia, Diphysa, Dalbergia, Machaerium*; **Clade 6**: *Kotschya, Humularia, Smithia, Cyclocarpa, Soemmeringia, Bryaspis, Aeschynomene, Ormocarpopsis, Peltiera, Ormocarpum, Pictetia, Diphysa*; **Clade 7**: *Centrosema, Clitoria, Dalbergiella*; **Clade 8**: *Ptycholobium, Mundulea, Chadsia, Tephrosia, Apurimacia, Lonchocarpus, Muellera, Derris, Paraderris, Fordia, Hesperothamnus, Piscidia, Gompholobium, Pongamiopsis, Erichsenia, Viminaria, Philenoptera, Leptoderris, Deguelia, Millettia, Galactia, Rhodopis, Dioclea, Canavalia, Aganope, Ostryocarpus*.
