# Supplementary material for: Reconstructing an historical pollination syndrome: keel flowers
Source: BMC Ecol Evol. 2022 Apr 12;22:45. doi: 10.1186/s12862-022-02003-y (PMC9004149; doi:10.1186/s12862-022-02003-y)
Supplement: Supplementary file 1 — Additional file 1. S1. Chronogram of Fabales. [file 12862_2022_2003_MOESM1_ESM.docx]

[Additional material, S15-Outgroup included chronogram of Fabales]

#NEXUS

begin trees;

tree TREE1 = [&R] ((Nothofagus_OF:115.56118,((Castanea_OF:3.990729,Quercus_OF:3.990729):93.317436,(((Juglans_OF:25.269898,Platycarya_OF:25.269898):57.420734,(Morella_OF:4.788365,Myrica_OF:4.788365):77.902267):6.638909,((Casuarina_OF:55.714811,Gymnostoma_OF:55.714811):29.736096,(Ticodendron_OF:82.905491,(Alnus_OF:19.612951,Betula_OF:19.612951):63.292541):2.545416):3.878635):7.978623):18.253015):11.3086,(((Anisophyllea_OC:30.327857,Combretocarpus_OC:30.327857):28.892575,((Corynocarpus_OC:43.007157,Coriaria_OC:43.007157):8.56474,((Cucumis_OC:23.137651,Bolbostemma_OC:23.137651):25.425469,((Hillebrandia_OC:18.704165,Begonia_OC:18.704165):23.125622,(Datisca_OC:37.04668,(Tetrameles_OC:17.607242,Octomeles_OC:17.607242):19.439438):4.783107):6.733332):3.008778):7.648534):58.000803,(((Fragaria_OR:53.999865,Prunus_OR:53.999865):37.708389,((Dirachma_OR:72.646634,((Spyridium_OR:26.658232,Rhamnus_OR:26.658232):43.917402,(Barbeya_OR:59.573746,(Elaeagnus_OR:40.294837,Hippophae_OR:40.294837):19.278909):11.001888):2.071):3.800029,((Zelkova_OR:15.778102,Ulmus_OR:15.778102):41.0973,((Urera_OR:33.048802,Urtica_OR:33.048802):15.630005,((Ficus_OR:28.057316,Morus_OR:28.057316):14.472117,(Cannabis_OR:14.848875,Humulus_OR:14.848875):27.680558):6.149374):8.196595):19.571262):15.26159):21.078746,((Bulnesia_OZ:101.90526,(Viola_OM:90.283604,(Oxalis_OO:80.661698,Celastrus_OCE:80.661698):9.621906):11.621656):9.797703,((Quillaja_Q:65.319226,((Cadellia_S:21.55171,Recchia_S:21.55171):22.740029,(Suriana_S:36.878173,(Stylobasium_S:17.185585,Guilfoylia_S:17.185585):19.692589):7.413566):21.027486):6.354097,((Moutabea_P:60.286556,(Xanthophyllum_P:53.566382,((Diclidanthera_P:12.4498,(Barnhartia_P:6.317104,Eriandra_P:6.317104):6.132697):33.733326,((Carpolobia_P:17.653047,Atroxima_P:17.653047):26.02606,(Bredemeyera_P:41.85525,(Comesperma_P:34.904015,((Monnina_P:24.442285,Polygala_P:24.442285):8.858211,(Salomonia_P:30.611351,(Muraltia_P:28.252665,Securidaca_P:28.252665):2.358686):2.689145):1.603519):6.951235):1.823857):2.504019):7.383256):6.720174):9.61125,(((Duparquetia_c:65.839365,((Adenolobus_c:36.043491,Cercis_c:36.043491):15.455552,((Griffonia_c:38.11938,(Bauhinia_c:25.394033,(Piliostigma_c:23.256412,Brenierea_c:23.256412):2.137621):12.725347):7.548361,(Gigasiphon_c:37.026495,(Tylosema_c:22.051274,(Barklya_c:18.177762,(Lysiphyllum_c:15.538601,(Lasiobema_c:7.668028,Phanera_c:7.668028):7.870574):2.639161):3.873512):14.975221):8.641246):5.831302):14.340322):1.978903,(((Daniellia_c:52.263165,(Brandzeia_c:16.201651,Neoapaloxylon_c:16.201651):36.061514):0.272524,(((Peltogyne_c:29.915952,(Guibourtia_c:23.608441,Hymenaea_c:23.608441):6.307511):5.249153,((Stemonocoleus_c:10.298836,Augouardia_c:10.298836):17.196578,(Eperua_c:10.350951,Eurypetalum_c:10.350951):17.144463):7.669692):3.431342,((Gilletiodendron_c:11.382135,Hylodendron_c:11.382135):17.150758,(Baikiaea_c:13.249337,(Sindora_c:8.62059,(Tessmannia_c:6.335836,(Copaifera_c:5.132653,(Sindoropsis_c:2.868536,Detarium_c:2.868536):2.264117):1.203183):2.284753):4.628747):15.283557):10.063554):13.939243):1.823012,(((Schotia_c:37.35087,(Goniorrhachis_c:23.997446,Barnebydendron_c:23.997446):13.353424):7.67009,((Hardwickia_c:6.324352,Colophospermum_c:6.324352):17.598432,((Gossweilerodendron_c:11.568976,Prioria_c:11.568976):7.357862,(Oxystigma_c:7.683219,Kingiodendron_c:7.683219):11.243618):4.995946):21.098175):8.77938,(((Neochevalierodendron_c:44.796552,Crudia_c:44.796552):4.20025,(Icuria_c:45.4445,Aphanocalyx_c:45.4445):3.552302):0.798597,((((Hymenostegia_c:8.121368,Micklethwaitia_c:8.121368):9.087481,(Lebruniodendron_c:12.603529,(Maniltoa_c:5.36349,Cynometra_c:5.36349):7.24004):4.60532):1.986333,(Amherstia_c:16.690853,(Tamarindus_c:14.95521,(Loesenera_c:11.538357,(Talbotiella_c:7.575796,Leonardoxa_c:7.575796):3.962561):3.416853):1.735643):2.504329):0.288751,(((Polystemonanthus_c:11.621819,Dicymbe_c:11.621819):3.616281,(Humboldtia_c:13.008777,(Normandiodendron_c:8.230549,(Scorodophloeus_c:5.997817,Zenkerella_c:5.997817):2.232732):4.778228):2.229323):3.350041,((Paramacrolobium_c:10.764634,Cryptosepalum_c:10.764634):6.959323,(((Macrolobium_c:7.758612,Heterostemon_c:7.758612):5.771007,((Paloveopsis_c:3.325255,Ecuadendron_c:3.325255):5.892422,(Brownea_c:6.338216,(Browneopsis_c:4.714247,(Elizabetha_c:2.754143,Paloue_c:2.754143):1.960105):1.623969):2.87946):4.311942):2.76241,(((Brodriguesia_c:5.678713,(Afzelia_c:2.534701,Intsia_c:2.534701):3.144012):7.980804,(Lysidice_c:7.608808,(Endertia_c:6.292559,Saraca_c:6.292559):1.316249):6.050709):2.132591,(((Gilbertiodendron_c:3.68275,Pellegriniodendron_c:3.68275):3.595872,(Librevillea_c:4.619257,(Didelotia_c:1.959549,Plagiosiphon_c:1.959549):2.659708):2.659365):3.483213,((Anthonotha_c:5.375477,(Oddoniodendron_c:4.955209,(Berlinia_c:3.758851,(Isoberlinia_c:2.34484,Englerodendron_c:2.34484):1.414011):1.196358):0.420267):2.684288,(Microberlinia_c:6.684068,(Brachystegia_c:5.774128,(Julbernardia_c:4.075201,(Tetraberlinia_c:1.240242,Bikinia_c:1.240242):2.834959):1.698927):0.90994):1.375696):2.702071):5.030273):0.499921):1.431928):0.864184):0.895792):30.311466):4.004941):0.558362):13.459567):0.770755,(((Dialium_c:27.954939,(Baudouinia_c:16.598729,Eligmocarpus_c:16.598729):11.356209):7.026591,(Poeppigia_c:29.661733,(Mendoravia_c:24.015886,((Storckiella_c:15.667827,(Petalostylis_c:12.74142,Labichea_c:12.74142):2.926407):5.474042,((Zenia_c:14.33688,(Martiodendron_c:12.665403,Koompassia_c:12.665403):1.671478):3.404669,(Dicorynia_c:14.504431,(Distemonanthus_c:4.354016,Apuleia_c:4.354016):10.150415):3.237118):3.40032):2.874017):5.645847):5.319797):32.719205,((((Ceratonia_c:48.589931,(Acrocarpus_c:45.079,Tetrapterocarpon_c:45.079):3.51093):8.464026,(Arcoa_c:34.100553,(Umtiza_c:24.197632,(Gleditsia_c:13.304125,Gymnocladus_c:13.304125):10.893507):9.902921):22.953404):4.106931,((((Chamaecrista_c:49.108525,(Senna_c:44.672518,Cassia_c:44.672518):4.436007):3.735193,(Melanoxylon_c:47.166732,(Batesia_c:20.236359,(Vouacapoua_c:8.803778,Pterogyne_c:8.803778):11.432581):26.930373):5.676986):2.831846,(((Mezoneuron_c:44.841179,(Moullava_c:42.619302,(Guilandina_c:10.101994,Pterolobium_c:10.101994):32.517308):2.221877):2.822166,(Caesalpinia_c:36.817742,((Haematoxylum_c:17.640628,Lophocarpinia_c:17.640628):3.422591,(Tara_c:10.365647,Coulteria_c:10.365647):10.697572):15.754522):10.845602):3.926516,((Cordeauxia_c:5.302258,Stuhlmannia_c:5.302258):35.479696,((Pomaria_c:22.150923,(Poincianella_c:14.050726,Erythrostemon_c:14.050726):8.100197):7.739038,((Libidibia_c:11.213499,Stahlia_c:11.213499):6.540499,(Balsamocarpon_c:14.756984,(Zuccagnia_c:13.296288,(Stenodrepanum_c:10.04981,Hoffmannseggia_c:10.04981):3.246478):1.460695):2.997014):12.135963):10.891994):10.807906):4.085705):1.677789,(Recordoxylon_c:55.279559,((((Moldenhawera_c:32.603634,Diptychandra_c:32.603634):4.006542,(Dimorphandra_c:12.076429,(Stachyothyrsus_c:9.340757,(Mora_c:7.65233,Burkea_c:7.65233):1.688427):2.735672):24.533747):9.589901,(Dinizia_m:41.999459,((Campsiandra_c:13.347894,(Arapatiella_c:8.609251,(Jacqueshuberia_c:6.227962,Tachigali_c:6.227962):2.38129):4.738642):20.657097,((Schizolobium_c:23.228763,(Bussea_c:11.228121,Peltophorum_c:11.228121):12.000642):4.457382,(Parkinsonia_c:14.842239,((Conzattia_c:3.301956,Cenostigma_c:3.301956):6.79708,(Colvillea_c:5.251063,(Delonix_c:2.680228,Lemuropisum_c:2.680228):2.570835):4.847973):4.743203):12.843906):6.318846):7.994468):4.200618):8.541399,((Pachyelasma_c:50.139741,Erythrophleum_c:50.139741):2.261804,((Pentaclethra_m:45.421633,Chidlowia_c:45.421633):2.970717,(((Pseudoprosopis_m:12.773091,(Xylia_m:5.428888,Calpocalyx_m:5.428888):7.344202):12.751211,(Sympetalandra_c:12.950762,(Adenanthera_m:3.32766,(Amblygonocarpus_m:1.580448,Tetrapleura_m:1.580448):1.747211):9.623103):12.573539):21.627326,((Piptadeniastrum_m:34.283823,(Elephantorrhiza_m:7.946644,Entada_m:7.946644):26.337179):11.878405,((Indopiptadenia_m:40.259435,Plathymenia_m:40.259435):4.337901,(((Leucaena_m:27.251479,(Neptunia_m:20.52249,Prosopidastrum_m:20.52249):6.728988):3.878459,(((Cylicodiscus_m:22.378681,(Fillaeopsis_m:20.741397,Newtonia_m:20.741397):1.637284):1.571185,(Alantsilodendron_m:9.922782,(Dichrostachys_m:7.016985,Calliandropsis_m:7.016985):2.905797):14.027084):4.16584,((Mimozyganthus_m:10.733057,Piptadeniopsis_m:10.733057):15.126027,((Xerocladia_m:9.938603,Prosopis_m:9.938603):16.186766,(Acacia_m:21.427808,(Viguieranthus_m:17.8485,(Schleinitzia_m:13.630522,(Desmanthus_m:10.810887,Kanaloa_m:10.810887):2.819635):4.217978):3.579308):4.697561):-0.266286):2.256622):3.014232):2.309296,((Parkia_m:13.411744,Anadenanthera_m:13.411744):16.117062,((Mimosa_m:23.059659,(Piptadenia_m:18.763763,(Adenopodia_m:6.574281,Thailentadopsis_m:6.574281):12.189482):4.295897):4.532383,((Microlobius_m:17.368329,(Pseudopiptadenia_m:15.292624,(Stryphnodendron_m:9.910437,(Parapiptadenia_m:4.536209,Hydrochorea_m:4.536209):5.374228):5.382187):2.075706):9.093594,((Albizia_m:19.243839,(Macrosamanea_m:11.756534,Blanchetiodendron_m:11.756534):7.487305):5.470735,((Calliandra_m:15.604811,Zapoteca_m:15.604811):5.608569,(Inga_m:17.398614,((Faidherbia_m:11.073982,(Lysiloma_m:8.90976,Cojoba_m:8.90976):2.164222):4.428219,((Pithecellobium_m:11.444033,(Havardia_m:9.988667,(Ebenopsis_m:4.211792,Leucochloron_m:4.211792):5.776875):1.455365):2.34398,(Paraserianthes_m:12.66695,(Zygia_m:11.200361,(Samanea_m:10.446869,(Abarema_m:8.47662,(Sphinga_m:10.88164,((Cathormion_m:5.508248,(Pseudosamanea_m:3.488429,Cedrelinga_m:3.488429):2.019819):0.323441,(Enterolobium_m:3.551154,(Chloroleucon_m:4.446689,(Pararchidendron_m:3.108312,Archidendron_m:3.108312):1.338376):-0.895534):2.280534):5.049951):-2.40502):1.97025):0.753491):1.46659):1.121063):1.714188):1.896413):3.814766):3.501193):1.74735):1.130119):1.936763):3.910428):11.158102):1.564892):0.989399):1.240723):4.009195):2.33993):0.538084):2.073795):3.807534):5.483276,(((Bobgunnia_pap:50.476478,(Candolleodendron_Pap:44.769222,Swartzia_Pap:44.769222):5.707256):5.733031,(Trischidium_Pap:28.640746,(Cyathostegia_Pap:11.365425,Ateleia_Pap:11.365425):17.275321):27.568763):7.678906,((((Angylocalyx_Pap:9.979021,Xanthocercis_Pap:9.979021):13.353645,(Castanospermum_Pap:7.721,Alexa-Pap:7.721):15.611666):31.115535,((Monopteryx_Pap:40.237002,(Taralea_Pap:25.576656,(Pterodon_Pap:8.687964,Dipteryx_Pap:8.687964):16.888691):14.660346):8.201616,(Dussia_pap:36.807956,((Myroxylon_pap:16.195024,(Myrospermum_pap:10.940063,Myrocarpus_pap:10.940063):5.254961):16.22925,(Amburana_Pap:26.80124,(Mildbraediodendron_Pap:13.288457,Cordyla_pap:13.288457):13.512783):5.623033):4.383682):11.630662):6.009584):8.964825,(((Amphimas_pap:17.515747,(Lecointea_pap:9.867249,(Exostyles_pap:8.461057,((Holocalyx_pap:6.90879,Zollernia_pap:6.90879):0.432299,(Harleyodendron_pap:4.912707,Uribea_pap:4.912707):2.428383):1.119968):1.406192):7.648498):40.80577,((Sweetia_pap:21.1757,(Luetzelburgia_Pap:13.603651,Vatairea_Pap:13.603651):7.572049):34.850549,((Sophora_pap:11.642903,Calia_pap:11.642903):37.704316,(Styphnolobium_pap:39.618363,(Cladrastis_pap:33.632929,Pickeringia_pap:33.632929):5.985434):9.728856):6.679029):2.295268):4.519126,((Aldina_pap:59.579577,(Vataireopsis_pap:24.476146,(Andira_Pap:10.321913,Hymenolobium_pap:10.321913):14.154233):35.103431):2.589692,((((Ormosia_pap:26.21162,(Spirotropis_pap:17.309273,Panurea_pap:17.309273):8.902347):32.989124,((Poecilanthe_Pap:32.10113,(Harpalyce_pap:27.448282,(Cyclolobium_Pap:22.47861,(Brongniartia_pap:16.735579,((Templetonia_pap:12.805481,Hovea_pap:12.805481):1.948188,(Lamprolobium_pap:11.434429,Plagiocarpus_pap:11.434429):3.31924):1.981909):5.743031):4.969672):4.652848):23.991528,(((Pericopsis_Pap:22.56723,Camoensia_Pap:22.56723):19.59487,(Diplotropis_pap:18.323412,Bowdichia_Pap:18.323412):23.838688):12.67182,(Clathrotropis_pap:53.181695,((Platycelyphium_pap:16.735763,(Dicraeopetalum_Pap:10.270523,Bolusanthus_Pap:10.270523):6.46524):34.294463,((((Baptisia_pap:5.162285,Thermopsis_pap:5.162285):18.627758,(Piptanthus_Pap:9.879535,Anagyris_pap:9.879535):13.910508):8.716378,((Ammopiptanthus_pap:23.835008,Ammodendron_pap:23.835008):0.887292,(Salweenia_Pap:26.592503,(Maackia_pap:18.017689,Euchresta_pap:18.017689):8.574814):-1.870204):7.784121):15.379526,((Cadia_pap:25.867598,(Cyclopia_Pap:20.540176,((Virgilia_Pap:14.567854,Calpurnia_pap:14.567854):4.120663,((Amphithalea_Pap:5.961957,Stirtonanthus_Pap:5.961957):7.606631,(Xiphotheca_Pap:9.933618,(Podalyria_Pap:6.483779,Liparia_Pap:6.483779):3.449839):3.63497):5.119928):1.851659):5.327422):19.236799,(((Crotalaria_pap:15.076197,Bolusia_pap:15.076197):13.836217,(Lotononis_pap:21.520665,((Wiborgia_pap:12.285362,(Rafnia_pap:9.068035,Aspalathus_pap:9.068035):3.217326):5.786607,((Lebeckia_pap:7.991563,Spartidium_pap:7.991563):5.828281,(Pearsonia_pap:7.266275,(Rothia_pap:5.148123,Robynsiophyton_pap:5.148123):2.118152):6.553569):4.252124):3.448697):7.391749):12.929462,((Melolobium_pap:23.525764,Dichilus_pap:23.525764):6.023808,((Anarthrophyllum_Pap:16.293342,Argyrolobium_pap:16.293342):5.139313,((Lupinus_pap:8.660922,Polhillia_pap:8.660922):9.989342,((Retama_pap:6.682313,Erinacea_pap:6.682313):8.906185,((Echinospartum_Pap:10.281121,(Cytisophyllum_pap:7.814215,Laburnum_pap:7.814215):2.466906):3.554044,((Argyrocytisus_Pap:6.735705,(Cytisus_pap:3.705745,Calicotome_Pap:3.705745):3.02996):4.995347,((Stauracanthus_Pap:7.021715,(Adenocarpus_Pap:5.593751,Ulex_pap:5.593751):1.427964):3.560339,(Genista_pap:9.064078,(Spartium_pap:4.759803,Petteria_pap:4.759803):4.304275):1.517976):1.148998):2.104113):1.753332):3.061768):2.78239):8.116917):12.292304):3.262521):2.781551):3.144279):2.151469):1.652225):1.258738):3.108086):2.284157,((((Errazurizia_pap:8.2945,Eysenhardtia_pap:8.2945):3.576356,(Apoplanesia_pap:7.289442,(Amorpha_pap:2.069171,Parryella_pap:2.069171):5.220271):4.581413):41.12508,((Daviesia_Pap:40.118788,Isotropis_pap:40.118788):7.033767,((Platylobium_Pap:22.443174,(Psorothamnus_pap:7.624065,Goodia_Pap:7.624065):14.819109):19.982287,((Phyllota_pap:20.717428,(Jacksonia_pap:19.525682,((Euchilopsis_pap:7.90296,Urodon_pap:7.90296):7.673823,(Leptosema_pap:18.121547,(Pultenaea_pap:11.722564,(Dillwynia_pap:5.407024,Aotus_pap:5.407024):6.315541):6.398982):-2.544764):3.948899):1.191746):12.733778,((Mirbelia_pap:29.56912,(Podolobium_pap:22.044429,((Callistachys_pap:12.214079,Chorizema_pap:12.214079):5.990879,(Eutaxia_pap:7.274736,Marina_pap:7.274736):10.930221):3.839472):7.524691):1.258637,((Dalea_pap:9.187467,(Stonesiella_pap:6.740257,Almaleea_pap:6.740257):2.44721):13.243749,(Latrobea_pap:22.071026,((Nemcia_pap:13.541816,Gastrolobium_pap:13.541816):2.958299,(Brachysema_pap:7.620003,Oxylobium_pap:7.620003):8.880111):5.570912):0.360189):8.396541):2.623449):8.974255):4.727094):5.843381):7.373373,(Acosmium_pap:55.901804,((Adesmia_Pap:43.788482,((Chaetocalyx_Pap:16.168089,Nissolia_Pap:16.168089):21.486049,(Poiretia_Pap:14.993287,(Amicia_Pap:11.118665,Zornia_Pap:11.118665):3.874622):22.66085):6.134344):9.407718,((Weberbauerella_Pap:45.136001,((Dalbergia_Pap:39.525865,Machaerium_Pap:39.525865):4.798706,(((Pictetia_Pap:9.875095,Diphysa_Pap:9.875095):1.947522,(Ormocarpum_Pap:6.539485,(Ormocarpopsis_Pap:3.711471,Peltiera_Pap:3.711471):2.828014):5.283132):31.31504,(Aeschynomene_Pap:30.217834,(Bryaspis_Pap:24.9451,((Cyclocarpa_pap:14.199216,Soemmeringia_pap:14.199216):6.726743,(Smithia_Pap:20.368741,(Kotschya_pap:17.319771,Humularia_pap:17.319771):3.048971):0.557218):4.019141):5.272734):12.919823):1.186914):0.81143):5.0141,((Riedeliella_Pap:26.750043,Discolobium_pap:26.750043):21.432128,(((Geoffroea_Pap:13.182779,Cascaronia_Pap:13.182779):28.126142,((Fissicalyx_Pap:14.548735,Fiebrigiella_Pap:14.548735):20.165756,(Chapmannia_pap:20.905648,(Arachis_pap:13.067617,Stylosanthes_pap:13.067617):7.838031):13.808842):6.59443):4.587614,(Platymiscium_Pap:41.340277,((Brya_Pap:11.671217,Cranocarpus_Pap:11.671217):28.219554,(Grazielodendron_Pap:37.507967,(Platypodium_Pap:28.132077,((Maraniona_Pap:11.825457,(Pterocarpus_Pap:11.944206,(Paramachaerium_Pap:7.628375,Etaballia_Pap:7.628375):4.315831):-0.118749):6.902287,((Centrolobium_Pap:7.984464,Inocarpus_Pap:7.984464):2.609296,(Tipuana_Pap:9.31237,Ramorinoa_Pap:9.31237):1.28139):8.133984):9.404333):9.37589):2.382805):1.449506):4.556257):2.285637):1.967929):3.0461):2.705604):4.467504):1.115592):0.204011,((Hypocalyptus_pap:57.55336,((Dalhousiea_pap:29.267091,Airyantha_Pap:29.267091):8.49762,(Baphia_Pap:23.451253,(Baphiopsis_Pap:16.423753,Leucomphalos_Pap:16.423753):7.0275):14.313458):19.788649):3.135118,(((Sesbania_pap:51.323522,(((Hippocrepis_pap:27.818332,(Coronilla_pap:4.983614,Securigera_pap:4.983614):22.834717):4.309203,((Ornithopus_pap:22.176117,Anthyllis_pap:22.176117):6.794451,(Lotus_pap:23.900203,(Dorycnium_pap:16.44946,Hammatolobium_pap:16.44946):7.450742):5.070365):3.156966):18.753711,((Hebestigma_pap:38.179771,(Lennea_pap:37.654743,(Poitea_pap:8.409093,Gliricidia_pap:8.409093):29.24565):0.525028):1.528325,(Robinia_pap:33.420026,((Sphinctospermum_pap:8.822241,Olneya_pap:8.822241):5.094378,(Coursetia_pap:11.224823,(Peteria_pap:9.496435,Genistidium_pap:9.496435):1.728388):2.691796):19.503407):6.28807):11.173149):0.442277):4.235375,((Callerya_pap:45.082418,Glycyrrhiza_pap:45.082418):5.038011,((Endosamara_pap:35.192906,Wisteria_pap:35.192906):13.08469,((Parochetus_pap:42.693384,(Galega_pap:39.428199,(Trigonella_pap:35.467655,((Trifolium_pap:24.651314,(Ononis_pap:22.23253,(Medica_pap:15.271816,Melilotus_pap:15.271816):6.960714):2.418784):4.699824,((Vicia_pap:16.013666,Lens_pap:16.013666):4.379178,(Lathyrus_pap:16.803031,(Vavilovia_pap:10.574376,Pisum_pap:10.574376):6.228655):3.589813):8.958294):6.116517):3.960544):3.265186):2.487015,((Cicer_pap:38.430997,(Erophaca_pap:34.849478,((Oxytropis_pap:21.202177,(Biserrula_pap:14.10287,(Barnebyella_pap:8.891227,Astragalus_pap:8.891227):5.211642):7.099307):8.033594,((Colutea_pap:19.554515,(Sphaerophysa_pap:9.093748,(Eremosparton_pap:2.277506,Smirnowia_pap:2.277506):6.816242):10.460768):5.248227,((Sutherlandia_pap:3.638201,Lessertia_pap:3.638201):19.160246,(Montigena_pap:12.918864,(Swainsona_pap:8.499484,(Clianthus_pap:5.541947,Carmichaelia_pap:5.541947):2.957537):4.41938):9.879582):2.004296):4.433028):5.613708):3.581519):4.340456,((Chesneya_pap:30.525561,(Tibetia_pap:19.069454,Gueldenstaedtia_pap:19.069454):11.456106):10.550454,((Caragana_pap:12.37231,(Halimodendron_pap:7.902933,Calophaca_pap:7.902933):4.469377):26.144045,(Oreophysa_pap:31.516439,(Alhagi_pap:28.816259,((Ebenus_pap:11.445776,Sulla_pap:11.445776):11.469881,(Taverniera_pap:17.865352,((Hedysarum_pap:13.059592,Sartoria_pap:13.059592):3.926819,(Onobrychis_pap:12.706144,(Corethrodendron_pap:8.272131,Eversmannia_pap:8.272131):4.434013):4.280267):0.878941):5.050305):5.900602):2.70018):6.999916):2.559659):1.695439):2.408946):3.097198):1.842832):5.438469):3.670474,((Geissaspis_Pap:47.833993,((Phylloxylon_Pap:32.511394,(Dunbaria_pap:12.795754,Indigofera_Pap:12.795754):19.71564):4.212592,((Microcharis_Pap:10.576039,Rhynchotropis_Pap:10.576039):19.1079,(Cyamopsis_Pap:16.156117,(Vaughania_pap:5.812825,Indigastrum_Pap:5.812825):10.343292):13.527821):7.040048):11.110006):9.494241,(((Dewevrea_Pap:14.750459,Platycyamus_Pap:14.750459):28.815414,(Sphaerolobium_pap:17.935308,(Xeroderris_Pap:13.71665,Craibia_Pap:13.71665):4.218658):25.630565):10.257758,((Dalbergiella_pap:45.046732,(Centrosema_pap:33.381208,Clitoria_Pap:33.381208):11.665525):8.356924,(Austrosteenisia_pap:52.055538,((Abrus_pap:48.016705,((Aganope_pap:23.377521,Ostryocarpus_pap:23.377521):20.410336,((Canavalia_pap:15.491407,(Dioclea_pap:11.966736,(Galactia_pap:7.609964,Rhodopis_pap:7.609964):4.356771):3.524671):24.221026,((Millettia_pap:21.399655,(Deguelia_pap:19.504628,(Philenoptera_pap:9.631729,Leptoderris_pap:9.631729):9.872899):1.895027):13.967417,(((Gompholobium_pap:19.610829,Pongamiopsis_pap:19.610829):4.6014,(Erichsenia_pap:5.325288,Viminaria_pap:5.325288):18.886941):5.152123,((Piscidia_pap:23.751651,(Fordia_pap:18.191243,Hesperothamnus_pap:18.191243):5.560408):2.844174,(((Lonchocarpus_pap:18.591087,Muellera_pap:18.591087):3.336072,(Derris_pap:14.74511,Paraderris_pap:14.74511):7.182048):4.211468,(Apurimacia_pap:20.185764,(Tephrosia_pap:13.393099,(Chadsia_pap:7.973149,(Ptycholobium_pap:5.456858,Mundulea_pap:5.456858):2.516291):5.419951):6.792665):5.952862):0.457199):2.768527):6.00272):4.345362):4.075423):4.228848):3.896736,((Muelleranthus_Pap:31.013514,(Disynstemon_pap:13.232713,(Ptychosema_Pap:8.98574,Aenictophyton_Pap:8.98574):4.246973):17.780801):19.447645,(Kunstleria_pap:45.483455,(Apios_pap:43.110427,((((Shuteria_pap:32.174322,(Hardenbergia_pap:13.339186,Kennedia_pap:13.339186):18.835136):5.517745,(Mucuna_pap:21.366423,(Cochlianthus_pap:8.726818,Craspedolobium_pap:8.726818):12.639606):16.325643):3.447362,(Eriosema_pap:38.140699,((Campylotropis_pap:17.789727,(Kummerowia_pap:10.900699,Lespedeza_pap:10.900699):6.889028):16.343705,((Desmodium_pap:9.925032,Hylodesmum_pap:9.925032):19.678365,(Phyllodium_pap:28.107486,((Dendrolobium_pap:6.951553,Tadehagi_pap:6.951553):17.641447,(Pseudarthria_pap:18.632667,(Alysicarpus_pap:15.451837,(Codoriocalyx_pap:9.44484,(Uraria_pap:4.018922,Christia_pap:4.018922):5.425918):6.006997):3.18083):5.960333):3.514486):1.49591):4.530035):4.007267):2.99873):0.689197,((Vandasina_pap:9.914038,Butea_pap:9.914038):29.785926,((Afgekia_pap:31.261543,(Adenodolichos_pap:25.298494,(Flemingia_pap:23.432423,(Cajanus_pap:19.612484,(Paracalyx_pap:12.364085,(Bolusafra_pap:8.649921,Rhynchosia_pap:8.649921):3.714164):7.248399):3.819939):1.866071):5.963049):6.648739,((Otoptera_pap:25.245486,(Psophocarpus_pap:16.853249,Erythrina_pap:16.853249):8.392237):10.008825,(((Strongylodon_pap:22.701631,Cologania_pap:22.701631):8.108324,((Sphenostylis_pap:21.218561,((Macrotyloma_pap:3.315072,Decorsea_pap:3.315072):12.059187,(Alistilus_pap:11.456752,(Nesphostylis_pap:6.102076,Dolichos_pap:6.102076):5.354676):3.917508):5.844302):4.777669,(Wajira_pap:23.606449,(((Lablab_pap:2.541858,Dipogon_pap:2.541858):14.376603,(Vatovaea_pap:10.819939,Spathionema_pap:10.819939):6.098522):1.934589,((Vigna_pap:10.579994,Physostigma_pap:10.579994):6.611098,((Phaseolus_pap:12.225373,(Ramirezella_pap:9.111499,Oxyrhynchus_pap:9.111499):3.113874):2.554623,((Strophostyles_pap:7.305688,Dolichopsis_pap:7.305688):3.416664,(Mysanthus_pap:7.766185,Macroptilium_pap:7.766185):2.956167):4.057644):2.411096):1.661958):4.753399):2.389781):4.813725):2.101345,(((Spatholobus_pap:14.161419,Dumasia_pap:14.161419):8.090329,((Pachyrhizus_pap:12.010835,Neorautanenia_pap:12.010835):8.251181,(Calopogonium_pap:13.205333,(Pseudeminia_pap:6.371447,Pseudovigna_pap:6.371447):6.833886):7.056683):1.989731):5.869145,(Neonotonia_pap:25.890719,((Amphicarpaea_pap:22.836047,(Pueraria_pap:18.868873,(Glycine_pap:10.037543,Ophrestia_pap:10.037543):8.831329):3.967174):1.078275,((Teramnus_pap:16.172143,Phylacium_pap:16.172143):3.744021,((Bituminaria_pap:10.074347,(Pediomelum_pap:6.533546,(Psoralidium_pap:4.564782,Rupertia_pap:4.564782):1.968764):3.540801):1.95359,(Hoita_pap:10.042093,((Psoralea_pap:7.879464,Cullen_pap:7.879464):2.03694,(Orbexilum_pap:8.213025,Otholobium_pap:8.213025):1.703379):0.125689):1.985844):7.888227):3.998158):1.976398):2.230174):4.790407):2.343011):2.655971):1.789683):2.128661):1.281802):2.373027):4.977705):1.452282):0.142097):1.348118):0.419975):3.504603):1.901138):1.459106):1.000434):0.480358):0.671374):0.572384):0.475389):2.755748):1.056572):0.888288):1.308782):1.775517):40.02964):1.084037):4.434235):9.648545);

end;
