# Supplementary material for: Reconstructing an historical pollination syndrome: keel flowers
Source: BMC Ecol Evol. 2022 Apr 12;22:45. doi: 10.1186/s12862-022-02003-y (PMC9004149; doi:10.1186/s12862-022-02003-y)
Supplement: Supplementary file 16 — Additional file 16. S16. Sources used to construct ancestral state analyses of Fabales. [file 12862_2022_2003_MOESM16_ESM.docx]

**Additional material S14- Sources used to construct ancestral state analyses of Fabales.**

**Adams CD, Proctor GR, Read RW. 1972.** *Flowering Plants of Jamaica.* University of the West Indies, Mona, Jamaica.

**Adema F. 1998.** The genera *Dioclea*, *Luzonia*, and *Macropsychanthus*. *Blumea*, **43**(1): 233-239.

**Adema F. 2000**. Notes on Malesian Fabaceae (Leguminosae-Papilionoideae), The Rare Genus *Burkilliodendron*. *Gard. Bull. (Singapore)*, **52**: 5-10.

**Adewale Db, Amazue Eu. 2018.** Floral maturation indices of African yam bean (*Sphenostylis* *stenocarpa* Hochst Ex. A. Rich) harms (Fabaceae). *Notulae Scientia Biologicae*, *10*(1), pp.102-106.

**Agnew ADQ. 1974.** *Upland Kenya Wildflowers*. Oxford University Press, London

**Ahmed ZU, Begum ZT, Hassan MA, Khondker M, Kabir SMH, Ahmad MATA, Ahmed ATA, Rahman AKA, Haque EU. 2008.** *Encyclopedia of flora and fauna of Bangladesh*. Asiatic Society of Bangladesh, Dhaka.

**Allen ON, Allen EK. 1981.** *The Leguminosae, a source book of characteristics, uses, and nodulation*. University of Wisconsin Press.

**Arthur JC. 1925.** *North American Flora, 7(10).* The New York Botanical Garden.

**Backer CA, Bakhuizen Van Den Brink RC.** **1963**. *Flora of Java (Spermatophytes only), Vol:1*. Noordhoff, Groningen, the Netherlands.

**Bajpai O, Srivastava AK, Kushwaha AK, Chaudhary LB. 2014**. Taxonomy of a monotypic genus *Indopiptadenia* (Leguminosae-Mimosoideae). *Phytotaxa*, **164**(2):061-078.

**Barneby RC. 1989**. A review of *Lecointea* (Fabaceae: Swartzieae) in South America. *Brittonia*, **41**(4): 351-355.

**Beyra Matos A, Lavin MT. 1999**. *Monograph of Pictetia (Leguminosae-Papilionoideae) and review of the Aeschynomeneae*. American Society of Plant Taxonomists.

**Black JM. 1948.** *Flora of South Australia. Part II. Casuarinaceae-Euphorbiaceae.*, (2nd ed.). South Australian Government Printing Division.

**Boatwright JS, Van Wyk BE. 2007.** A new species of *Rafnia* (Crotalarieae, Fabaceae). *South African Journal of Botany*, **73**(3): 471-473.

**Boatwright JS, Van Wyk BE. 2009.** A revision of the African genus *Robynsiophyton* (Crotalarieae, Fabaceae). *South African Journal of Botany*, **75**(2): 367-370.

**Bond P, Goldblatt P. 1984.** *Plants of the Cape Flora: a descriptive catalogue.*, (Suppl. 13). Kirstenbosch.

**Boulos L. 2005.** *Flora of Egypt*, Vol. 1. Cairo: Al Hadara Publishing.

**Brandis D. 1906.** *Indian Trees: an account of trees, shrubs, woody climbers, bamboos and palms indigenous or commonly cultivated in the British Indian Empire*. Constable.

**Brenan JPM, Exell AW, Fernandes A, Wild H. 1970** *Flora Zambesiaca. Volume 3, Part 1*. Crown Agents, London, and Royal Botanic Gardens, Kew.

**Brenan JPM. 1986.** The Genus *Adenopodia* (Leguminosae). *Kew Bulletin*, 73-90.

**Brown FBH. 1935.** *Flora of southeastern Polynesia.* Honolulu, Hawai.

**Camargo RA, de Azevedo Tozzi AMG. 2014.** A synopsis of the genus *Deguelia* (Leguminosae, Papilionoideae, Millettieae) in Brazil. *Brittonia*, **66**(1): 12-32.

**Compton RH***.* **1976**. *The flora of Swaziland, Supplementary Vol. 11*. Trustees of the National Botanic Gardens of South Africa.

**Cardoso D, de Lima HC, Rodrigues RS, de Queiroz LP, Pennington RT, Lavin M.** **2012**. The realignment of *Acosmium* sensu stricto with the Dalbergioid clade (Leguminosae: Papilionoideae) reveals a proneness for independent evolution of radial floral symmetry among early-branching papilionoid legumes. *Taxon*, **61**(5): 1057-73.

**Cardoso D, De Queiroz LP, Pennington RT, De Lima HC, Fonty É, Wojciechowski MF, Lavin M. 2012.** Revisiting the phylogeny of papilionoid legumes: New insights from comprehensively sampled early‐branching lineages. *American Journal of Botany*, **99**(12): 1991-2013.

**Cardoso D, Pennington RT, de Queiroz LP, Boatwright JS, Van Wyk BE, Wojciechowski MF, Lavin M. 2013.** Reconstructing the deep-branching relationships of the papilionoid legumes. South African Journal of Botany, **89**: 58-75.

**Cardoso DBOS, de Queiroz LP, de Lima HC. 2014.** A taxonomic revision of the South American papilionoid genus *Luetzelburgia* (Fabaceae). *Botanical journal of the Linnean Society*, **175**(3): 328-375.

**Cardoso D, de Lima HC, Rodrigues RS, de Queiroz LP, Pennington RT, Lavin, M., 2012.** The realignment of *Acosmium* sensu stricto with the Dalbergioid clade (Leguminosae: Papilionoideae) reveals a proneness for independent evolution of radial floral symmetry among early-branching papilionoid legumes. *Taxon*, **61**(5): 1057-1073.

**Cardoso D, São-Mateus WM, da Cruz DT, Zartman CE, Komura DL, Kite G, Prenner G, Wieringa JJ, Clark A, Lewis G, Pennington RT. 2015.** Filling in the gaps of the papilionoid legume phylogeny: the enigmatic Amazonian genus *Petaladenium* is a new branch of the early-diverging Amburaneae clade. *Molecular Phylogenetics and Evolution*, **84**: 112-124.

**Castro S, Silveira P, COUTINHO AP, Figueiredo E. 2005.** Systematic studies in *Tylosema* (Leguminosae). *Botanical Journal of the Linnean Society*, **147**(1): 99-115.

**Chandler GT, Crisp MD, Cayzer LW, Bayer RJ. 2002.** Monograph of *Gastrolobium* (Fabaceae: Mirbelieae). *Australian Systematic Botany*, **15**(5): 619-739.

**Citerne HL, Pennington RT, Cronk QC. 2006.** An apparent reversal in floral symmetry in the legume *Cadia* is a homeotic transformation. *Proceedings of the National Academy of Sciences*, **103**(32): 12017-12020.

**Contreras-Jimenez JL, Sotuyo S, Calvillo-Canadell L, Lewis GP. 2017.** *Erythrostemon sousanus* (Leguminosae: Caesalpinioideae), a new species from the Río Papagayo Basin in Guerrero, México. *Phytotaxa*, **308**(2): 289-294.

**Corner EJH. 1940.** *Wayside trees of Malaya, Vols. I & II*. United Selangor Press, Kuala Lumpur, Malaysia.

**Correll DS, Correll HB. 1982.** *Flora of the Bahama Archipelago.* Gantner Verlag KG.

**Cowan RS. 1967**. *Swartzia* (Leguminosae, Caesalpinioideae Swartzieae). *Flora Neotropica*, **1**: 1-228.

**Cowan RS. 1975.** A monograph of the genus *Eperua* (Leguminosae: Caesalpinioideae). *Smithsonian Contributions to Botany*.

**Cowan RS. 1979.** *Harleyodendron*, a new genus of Leguminosae (Swartzieae). *Brittonia*, **31**(1): 72-78.

**Cronquist A, Holmgren AH, Holmgren NH, Reveal JL, Holmgren PK. 1977.** *Intermountain flora. Vascular plants of the Intermountain West, USA Vol:3, part B: Fabales by Barneby RC. The monocotyledons.* Intermountain flora. Vascular plants of the Intermountain West, USA Volume six. The monocotyledons.

**Curtis WM, Morris DI. 1956.** *The student's flora of Tasmania. Part I. Gymnospermae. Angiospermae: Ranunculaceae to Myrtaceae.* University of Tasmania, Hobart.

**Dassanayake MD, Fosberg FR. 1980**. *A Revised Handbook to the Flora of Ceylon* Vol. 1. CRC PressI Llc.

**Davis PH. 1970.** *Flora of Turkey. Vol. 3.* Edinburgh University Press.

**Degener O. 1933.** *The New Illustrated Flora of the Hawaiian Islands: Flora Hawaiiensis Books 1-4.* New York.

**de Freitas Mansano V, Lewis GP. 2004.** A revision of the genus *Exostyles* Schott (Leguminosae: Papilionoideae). *Kew Bulletin*, 521-529.

**de Freitas Mansano V, Bittrich V, de Azevedo T, Ana MG, de Souza, A.P. 2004.** Composition of the Lecointea clade (Leguminosae, Papilionoideae, Swartzieae), a re-evaluation based on combined evidence from morphology and molecular data. *Taxon*, **53**(4): 1007-1018.

**de la Estrella M, Aedo C, Mackinder B, Velayos M. 2010.** Taxonomic Revision of *Daniellia* (Leguminosae: Caesalpinioideae). *Systematic Botany*, **35**(2): 296-324.

**de la Estrella M, Devesa JA, Wieringa JJ. 2012**. A morphological re-evaluation of the taxonomic status of the genus *Pellegriniodendron* (Harms) J. Léonard (Leguminosae–Caesalpinioideae–Detarieae) and its inclusion in *Gilbertiodendron* J. Léonard. *South African Journal of Botany*, **78**: 257-265.

**de Lima HC. 1988.** A New Species of *Hymenolobium* (Leguminosae-Papilionoideae) From Central America. *Annals of the Missouri Botanical Garden*, **75**(3): 1145-1147.

**de Queiroz LP, Lewis GP, Allkin R. 1999.** A revision of the genus *Moldenhawera* Schrad. (Leguminosae-Caesalpinioideae). *Kew Bulletin*, 817-852.

**de Queiroz LP, São-Mateus W, Delgado-Salinas A, Torke BM, Lewis GP, Dorado Ó, Ardley JK, Wojciechowski MF, Cardoso D. 2017.** A molecular phylogeny reveals the Cuban enigmatic genus *Behaimia* as a new piece in the Brongniartieae puzzle of papilionoid legumes. *Molecular Phylogenetics and Evolution*, ***109***: 191-202.

**du Puy DJ, Labat JN, Rabevohitra R, Villiers JF, Bosser J, Moat J. 2002.** *The Leguminosae of Madagascar.* Royal Botanic Gardens, Kew.

**Eriksen B. 1993**. A revision of *Monnina* subg. Pterocarya (Polygalaceae) in northwestern South America. *Annals of the Missouri Botanical Garden*, 191-207.

**Eriksen B. 1993**. Phylogeny of the Polygalaceae and its taxonomic implications. *Plant Systematics and Evolution*, **186**(1-2): 33-55.

**Eriksen B, Stahl B, Persson C.** *Flora of Ecuador (65), Vol: 102, Polygalaceae.* *Edited by* G. Harling and L. Andersson. Botanical Institute, University of Goteborg, Sweden.

**Exell AW, Wild H. 1970** *Flora Zambesiaca. Volume 1, Part 1.* Crown Agents, London, and Royal Botanic Gardens, Kew.

**Filipe de Portugal ST, Lewis GP, Hawkins, J.A., 2010**. A revision of the South American genus *Apuleia* (Leguminosae, Cassieae). *Kew Bulletin*, **65**(2): 225-232.

**Ford AJ, Halford DA, Van Der Merwe M, Mathieson MT. 2017**. A revision of the tropical white-flowered species of *Comesperma* (Polygalaceae) in Australia. *Australian Systematic Botany*, **30**(2): 159-182.

**Forest F, Manning JC. 2006.** Evidence for inclusion of South African endemic *Nylandtia* in *Muraltia* (Polygalaceae). *Systematic Botany*, **31**(3): 525-532.

**Fortunato RH, De Queiroz LP, Lewis GP. 1996.** Lackeya, a new genus in tribe Phaseoleae subtribe Diocleinae (Leguminosae: Papilionoideae) from North America. *Kew Bulletin*, 365-370.

**Fosberg FR, Renvoize SA. 1919.** *Flora of Aldabra: with notes on the flora of the neighbouring islands.* Bulletin of Miscellaneous Information, Royal Botanic Gardens, Kew.

**Gagnon E, Bruneau A, Hughes CE, de Queiroz LP, Lewis GP. 2016.** A new generic system for the pantropical Caesalpinia group (Leguminosae). *PhytoKeys*, **71**: 1-160.

**Gélvez-Zúñiga I, Neves AC, Teixido AL, Fernandes GW. 2018**. Reproductive biology and floral visitors of *Collaea cipoensis* (Fabaceae), an endemic shrub of the rupestrian grasslands. *Flora*, **238**: 129-137.

**Gentry AH, Vasquez R. 1993.** *A field guide to the families and genera of woody plants of northwest South America (Colombia, Ecuador, Peru): with supplementary notes on herbaceous taxa.* The University of Chicago Press, Chicago.

**Goldblatt P, Manning J. 2000.** *Cape plants: a conspectus of the Cape flora of South Africa*. National Botanical Institute.

**Grossi MA, Funes G. 2011.** Biología de especies australes: *Apurimacia dolichocarpa* (Griseb.) Burkart (Papilionoideae-Leguminosae). Tomo **36**(2): 47-52.

**Grubov VI. 2003.** *Plants of Central Asia-Plant Collection from China and Mongolia, Vol. 8a: Leguminosae*. CRC Press.

**Gunn CR, Norman EM, Lassetter JS. 1980.** *Chapmannia floridana* Torrey & Gray (Fabaceae). *Brittonia*, **32**(2): 178-185.

**Hitchcock CL, Cronquist A. 1973.** *Flora of the Pacific Northwest: an illustrated manual*. University of Washington Press.

**Hutchinson J, Dalziel JM. 1954.** Flora of west tropical Africa. *Flora of West Tropical Africa. The British West African Colonies, British Cameroons, the French and Portuguese Colonies south of the Tropic of Cancer to Lake Chad, and Fernando Po.*, *1*(Part II). Royal Botanic Gardens, Kew.

**Hedberg I, Edwards S. 1989.** *Flora of Ethiopia, Vol. 3, Pittosporaceae to Araliacae.* The National Herbarium, Addis Ababa University.

**Heenan PB. 1998.** *Montigena* (Fabaceae), a new genus endemic to New Zealand. *New Zealand Journal of Botany*, **36**(1): 41-51.

**Herendeen PS. 1995.** Phylogenetic relationships of the tribe Swartzieae. In *Advances in Legume Systematics, part 7, Phylogeny*. Royal Botanic Gardens, Kew, 123-132.

**Hernández HM, Guinet P. 1990.** *Calliandropsis*: a new genus of Leguminosae: Mimosoideae from Mexico. *Kew Bulletin*, 609-620.

**Hooker JD.** 1872. *Flora of British India,* Vol. II. Reeve & Co., London.

**Huang TC. 1996.** Flora of Taiwan, second ed., vol. 2. Editorial Committee of the Flora of Taiwan, Taipei.

**Ireland H, Pennington RT, Preston J. 2000**. Molecular systematics of the Swartzieae. In: Herendeen PS and Bruneau A. eds. Advances in Legume Systematics, Part 9, Royal Botanic Gardens, Kew, 217–31.

**Ireland HE. 2007.** Taxonomic changes in the South American genus *Bocoa* (Leguminosae-Swartzieae): reinstatement of the name *Trischidium*, and a synopsis of both genera. *Kew Bulletin*, 333-349.

**Irwin HS, Arroyo MTK. 1972.** A New species of *Periandra* (Leguminosae: Lotoideae) from the Planalto of Brazil. *Brittonia*, **24**(3): 327-329.

**Kirkbride JH, Wiersema JH. 1997.** *Bobgunnia*, a new African genus of tribe Swartzieae (Fabaceae, Faboideae). *Brittonia*, **49**(1): 1-23.

**Kitamura S. 1960.** *Flora of Afghanistan. Results of the Kyoto University Scientific Expedition to the Karakoram and Hindu-kush, Vol. II*, *2*. Kyoto University

**Klitgård BB, Forest F, Booth TJ, Saslis-Lagoudakis CH. 2013**. A detailed investigation of the *Pterocarpus* clade (Leguminosae: Dalbergieae): *Etaballia* with radially symmetrical flowers is nested within the papilionoid-flowered *Pterocarpus*. *South African Journal of Botany*, **89**: 128-142.

**Kochanovski FJ, Paulino JV, Teixeira SP, Tozzi AMGDA, Mansano VDF. 2018.** Floral development of *Hymenaea verrucosa*: an ontogenetic approach to the unusual flower of Fabaceae subfamily Detarioideae. *Botanical Journal of the Linnean Society*, **187**(1): 46-58.

**Koenen EJ, Ojeda DI, Steeves R, Migliore J, Bakker FT, Wieringa JJ, Kidner C, Hardy OJ, Pennington RT, Bruneau, Hughes CE. 2019.** Large‐scale genomic sequence data resolve the deepest divergences in the legume phylogeny and support a near‐simultaneous evolutionary origin of all six subfamilies. New Phytol. 225: 1355–69

**Koeppen R, Iltis HH. 1962.** Revision of *Martiodendron* (Cassieae, Caesalpiniaceae). *Brittonia*, **14**(2): 191-209.

**Koeppen RC. 1967**. Revision of *Dicorynia* (Cassieae, Caesalpiniaceae). *Brittonia*, **19**(1): 42-61.

**Kubitzki EK. 1990.** In: *The families and genera of vascular plants*, Vol. 9. Berlin: Springer.

**Labat JN, Du Puy DJ. 1996.** Two new species of *Ormocarpopsis* R. Viguier and a new combination in *Ormocarpum* P. Beauvois (Leguminosae-Papilionoideae) from Madagascar. *Novon*, 54-58.

**Lanjouw J, Stoffers AL. 1932-1939.** *Flora of Suriname, Vol:3, Part:1.* Royal Tropical Institute, Amsterdam.

**Lavin M. 1993.** Biogeography and systematics of Poitea (Leguminosae): inferences from morphological and molecular data. *Systematic Botany Monographs*, 1-87.

**Lavin M, Sousa S. 1995.** Phylogenetic systematics and biogeography of the tribe Robinieae (Leguminosae). *Systematic Botany Monographs*, 1-165.

**Lavin M, Wojciechowski MF, Richman A, Rotella J, Sanderson MJ, Matos, AB. 2001.** Identifying Tertiary radiations of Fabaceae in the Greater Antilles: alternatives to cladistic vicariance analysis. *International Journal of Plant Sciences.* **162**: S53-76.

**Lebrun JP, Stork AL. 2008.** *Tropical African Flowering Plants: Ecology and Distribution. Volume 4: Fabaceae (Desmodium-Zornia)*. Conservatoire et Jardin Botaniques de la Ville de Genève.

**Leistner OA. 2000.** *Seed plants of southern Africa: families and genera*. National Botanical Institute.

**Leite VG, Teixeira SP, Mansano VF, Prenner G. 2014**. Floral development of the early-branching papilionoid legume Amburana cearensis (Leguminosae) reveals rare and novel characters. *International Journal of Plant Sciences*, **176**(1): 94-106.

**Leite VG, Mansano VF, Teixeira SP.2014.** Floral ontogeny in Dipterygeae (Fabaceae) reveals new insights into one of the earliest branching tribes in papilionoid legumes. *Botanical Journal of the Linnean Society.* **174**(4): 529-50.

**León-de la Luz JL, Pérez-Navarro JJ, Domínguez-Cadena R. 2002**. Two new *Marina* (Leguminosae) from the southern Baja California peninsula, Mexico. *Brittonia*, **54**(2): 72-77.

**LeRoux MM. Van Wyk BE. 2009.** A revision of *Lebeckia* sect. Lebeckia: The *L. pauciflora* and *L. wrightii* groups (Fabaceae, Crotalarieae). *South African Journal of Botany*, **75**(1): 83-96.

**Lewis GP, Salinas AD. 1994.** *Mysanthus*, a new genus in tribe Phaseoleae (Leguminosae: Papilionoideae) from Brazil. *Kew Bulletin*, 343-351.

**Thailentadopsis Kostermans (Leguminosae: Mimosoideae: Ingeae) ResurrectedThailentadopsis Kostermans (Leguminosae: Mimosoideae: Ingeae) ResurrectedLewis G, Schrire B. 2003.** *Thailentadopsis* Kostermans (Leguminosae: Mimosoideae: Ingeae) Resurrected. *Kew Bulletin,* **58**(2): 491-494. doi:10.2307/4120634

**Lewis GP. 2005.** *Legumes of the World*. Royal Botanic Gardens Kew.

**Lewis GP, de Queiroz LP. 2010**. *Moldenhawera* intermedia (Leguminosae: Caesalpinioideae), a new species from the Brazilian state of Bahia. *Kew Bulletin*, **65**(2): 205-207.

**Lewis GP, Siqueira GS, Banks H, Bruneau A. 2017**. The majestic canopy-emergent genus *Dinizia* (Leguminosae: Caesalpinioideae), including a new species endemic to the Brazilian state of Espírito Santo. *Kew bulletin*, **72**(3): 48.

**Leys R, Hogendoorn K. 2008.** Correlated evolution of mating behaviour and morphology in large carpenter bees (*Xylocopa*). Apidologie.**39**(1): 119-32.

**Li HL. 1963.** *Woody Flora of Taiwan.* Morris Arboretum, The University of Pennsylvania.

**Lima HCD, Vaz AMSDF. 1984.** Revisão taxonômica do gênero *Riedeliella* Harms (Leguminosae-Faboideae). *Rodriguésia*, ***36***(58), 9-16.

**Lock JM. 2006.** A new species of *Scorodophloeus* (Leguminosae: Caesalpinioideae: Detarieae) from Mozambique. *Kew Bulletin*, 257-259.

**Lombardi JA. 2002**. *Martiodendron* *fluminense* (Leguminosae, Caesalpinioideae), a new species from the Atlantic coast rainforest of Brazil. *Brittonia*, **54**(4): 327-330.

**Lorence DH, Wood KR. 1994**. *Kanaloa*, a new genus of Fabaceae (Mimosoideae) from Hawaii. *Novon*, 137-145.

**LPWG, 2017.** A new subfamily classification of the Leguminosae based on a taxonomically comprehensive phylogeny. *Taxon*, **66**(1): 44-77.

**Mansano VF, Bittrich V, De Azevedo T, Ana MG, De Souza AP. 2004.** Composition of the *Lecointea* clade (Leguminosae, Papilionoideae, Swartzieae), a re-evaluation based on combined evidence from morphology and molecular data. *Taxon* 53(4): 1007-18.

**Marazzi B, Ané C, Simon MF, Delgado‐Salinas A, Luckow M, Sanderson MJ. 2012.** Locating evolutionary precursors on a phylogenetic tree. *Evolution* 66(12): 3918-3930.

**Mc Carthy. 1998.** *Flora of Australia, Volume 12, Mimosaceae (excl. Acacia), Caesalpiniaceae.* Australian Government Publishing Service, Canberra.

**McGregor RL, Barkley TM, Brooks RE, Schofield EK.** **1986.** *Flora of the Great Plains.* University Press of Kansas.

**McMahon M, Hufford L. 2004.** Phylogeny of *Amorpheae* (Fabaceae: Papilionoideae). *American Journal of Botany*, **91**(8): 1219-1230.

**Meikle RD. 1977.** *Flora of Cyprus. Vol. 1.* Royal Botanic Gardens, Kew.

**Melo MMRF, Barros F, Chiea SAC, Kirizawa M, Jung-Mendaçolli SL, Wanderley MGL.** **2000.** *Flora Fanerogâmica da Ilha do Cardoso. Vol.7.* Instituto de Botânica, São Paulo.

**Miège MN. 1978.** *Cordeauxia* Edulis—A caesalpiniaceae of arid zones of east Africa. *Economic Botany*, **32**(3): 337-345.

**Milne-Redhead E, Polhill RM. 1970.** *Flora of Tropical East Africa, Leguminosae (Part 3), Subfamily Papilionoideae (1).* Royal Botanic Gardens, Kew, UK.

**Milne-Redhead E, Polhill RM. 1970.** *Flora of Tropical East Africa, Leguminosae (Part 4), Subfamily Papilionoideae (2).* Royal Botanic Gardens, Kew, UK.

**Moore DM. 1983.** *Flora of Tierra del Fuego.* Missouri Botanical Garden, Missouri.

**Mota M, Pastore JFB. 2018.** Two new species of *Bredemeyera* (Polygalaceae) from Brazil. *Phytotaxa*, **351**(2): 171-175.

**Moteetee A, Van Wyk BE. 2006.** A revision of the genus *Melolobium* (Genisteae, Fabacaeae). *South African Journal of Botany*, **72**(1): 51-98.

**Moura TM, Lewis GP, Tozzi AMGA. 2016.** A revision of the South American genus *Platycyamus* Benth. (Leguminosae). *Kew bulletin*, **71**(1): 9.

**Munz PA, Keck DD. 1968.** *A California Flora and Supplement.* Berkeley: University of California Press.

**Neill DA. 1998.** *Ecuadendron* (Fabaceae: Caesalpinioideae: Detarieae): a new arborescent genus from western Ecuador. *Novon*, 45-49.

**Nemoto T, Murata J. 2013.** A new record of *Desmodiastrum parviflorum* (Leguminosae) from Myanmar. *J. Jpn. Bot*, **88**: 21-29.

**Nielsen IC. 1985.** *Flora of Thailand, Vol:4, Part: 2.* Royal Forest Department, Bangkok.

**Ohwi J. 1965.** *Flora of Japan.* Smithsonian Institution, Washington, DC.

**Ojeda DI, Koenen E, Cervantes S, de la Estrella M, Banguera-Hinestroza E, Janssens SB, Migliore J, Demenou BB, Bruneau A, Forest F, Hardy OJ. 2019.** Phylogenomic analyses reveal an exceptionally high number of evolutionary shifts in a florally diverse clade of African legumes. *Molecular Phylogenetics and Evolution* 137: 156-67.

**Palacios RA, Hoc PS. 2001.** Three new species of Prosopidastrum (Mimosaceae) from Argentina. *Novon*, 79-87.

**Parker N. 1918.** *A Forest Flora for the Punjab with Hazara and Delhi.* 1918. *Edited by* R. N. Parker. Lahore Government Printing Press West Pakistan.

**Parker T. 2008.** *Trees of Guatemala.* Tree Press, Austin.

**Pastore JFB. 2010.** A synopsis, new combinations, and synonyms in Acanthocladus (Polygalaceae). *Novon: A Journal for Botanical Nomenclature*, **20**(3): 317-325.

**Pendry CA. 2010.** Epirixanthes compressa Pendry, a new mycoheterotrophic species of Polygalaceae from Thailand. *Thai Forest Bulletin (Botany)*, **38**: 184-186.

**Pennington RT, Klitgaard BB, Ireland HE, Lavin M. 2000.** New insights into floral evolution of basal Papilionoideae from molecular phylogenies. *Advances in Legume Systematics, part*, *9*, 233-248.

**Pennington RT, Lavin M, Ireland H, Klitgaard B, Preston J, Hu J-M. 2001.** Phylogenetic relationships of basal papilionoid legumes based upon sequences of the chloroplast *trnL* intron. *Systematic Botany* 26: 537-56.

**Perera MJ, Alves M. 2018.** Flora da Usina São José, Igarassu, Pernambuco: Polygonaceae. *Rodriguésia*, **69**(2): 465-476.

**Phillips EP. 1951.** *The genera of South African flowering plants.* Cape Times Ltd., Govt. Printers.

**Piper CV. 1924.** The genus *Oxyrhynchus Brandegee*. *Journal of the Washington Academy of Sciences*, **14**(2): 46-49.

**Polunin O, Stainton A. 1984.** *Flowers of the Himalaya*. Oxford University Press.

**Prenner G, Klitgaard BB. 2008**. Towards unlocking the deep nodes of Leguminosae: floral development and morphology of the enigmatic *Duparquetia orchidacea* (Leguminosae, Caesalpinioideae). *American Journal of Botany*, **95**(11): 1349-1365.

**Prenner G, Cardoso D, Zartman CE, de Queiroz LP. 2015**. Flowers of the early‐branching papilionoid legume *Petaladenium urceoliferum* display unique morphological and ontogenetic features. *American journal of botany*, **102**(11): 1780-1793.

**Poorter L, Bongers F, Kouame FN, Hawthorne WD.** **2004**. *Biodiversity of West African forests: an ecological atlas of woody plant species*. CABI.

**Pulle A. 1976.**  *Flora of Suriname, Vol:2, Part:2.* Royal Tropical Institute, Amsterdam.

**Queiroz LD. 2006**. New species and new combinations in *Phanera* Lour. *Caesalpinioideae: Cercideae) from the caatinga biome. Neodiversity*, **1**: 6-10.

**Ramos G, de Lima HC, Prenner G, de Queiroz LP, Zartman CE, Cardoso, D., 2016.** Molecular systematics of the Amazonian genus *Aldina*, a phylogenetically enigmatic ectomycorrhizal lineage of papilionoid legumes. *Molecular Phylogenetics and Evolution*, **97**: 11-18.

**Reyes E, Sauquet H, Nadot S. 2016.** Perianth symmetry changed at least 199 times in angiosperm evolution. *Taxon* 65(5): 945-64.

**Ribeiro PG, Luckow M, Lewis GP, Simon MF, Cardoso D, de Souza ÉR, Conceicao Silva AP, Jesus MC, dos Santos FA, Azevedo V, de Queiroz LP. 2018.** *Lachesiodendron*, a new monospecific genus segregated from *Piptadenia* (Leguminosae: Caesalpinioideae: mimosoid clade): Evidence from morphology and molecules. *Taxon*, **67**(1): 37-54.

**Rico MDL. 1994**. Four new species of *Zygia* (Leguminosae: Mimosoideae). *Kew Bulletin*, 547-554.

**Rudd VE. 1981.** Two new species of *Paramachaerium* (Leguminosae) and a brief résumé of the genus. *Brittonia*, **33**(3): 435-440.

**Russo L, Memmott J, Montoya D, Shea K, Buckley YM. 2014.** Patterns of introduced species interactions affect multiple aspects of network structure in plant–pollinator communities. *Ecology* 95(10): 2953-63.

**Rydberg PA. 1965.**  *Flora of the Prairies and Plains of Central North America*. New York Botanical Garden, USA.

**Rydberg PA. 1917.** *Flora of the Rocky Mountains and adjacent plains, Colorado, Utah, Wyoming, Idaho, Montana, Saskatchewan, Alberta, and Neighboring Parts of Nebraska, South Dakota, and British Columbia.* New York Botanical Garden, USA.

**Saldivia P, Faundez L. 2014.** *Weberbauerella chilensis* (Fabaceae: Papilionoideae), a new species from the Atacama Desert, Chile. *Phytotaxa*, **156**(1): 41-46.

**Salinas AD, Johnston MC. 1984.** A new species of *Myrospermum* (Leguminosae: Papilionoideae) from northeastern Mexico. *Systematic botany*, 356-358.

**Santisuk T, Larsen K. 2001.** *Flora of Thailand, Vol:7, Part: 3.* Royal Forest Department, Bangkok.

**São-Mateus W, Paganucci de Queiroz L, Jardim JG, Cardoso DB. 2018**. *Harpalyce riparia* (Leguminosae, Papilionoideae), a New Species from the Campos Rupestres of the Chapada Diamantina in Bahia, Brazil. *Systematic botany*, **43**(1): 206-211.

**Sauquet H, von Balthazar M, Magallón S, Doyle JA, Endress PK, Bailes EJ, de Morais EB, Bull-Hereñu K, Carrive L, Chartier M, Chomicki G. et al. 2017.** The ancestral flower of angiosperms and its early diversification. *Nat Commun* 8: 16047.

**Schrire BD. 2000.** A synopsis of the genus *Philenoptera* (Leguminosae-Millettieae) from Africa and Madagascar. *Kew Bulletin*, 81-94.

**Scoggan HJ.** *The Flora of Canada, Part 3.* National Museums of Canada.

**Seleme EP, Lewis GP, Stirton CH, Sartori ALB, Mansano VF. 2015.** A Taxonomic review and a new species of the South American woody genus *Amburana* (Leguminosae, Papilionoideae). *Phytotaxa*, **212**(4): 249-263.

**Silva MJ, Tozzi AMGA. 2010.** *Leguminosae-subfamília Caesalpinioideae.* *Flora Fanerogâmica da Ilha do Cardoso. Vol. 15.* Instituto de Botânica, São Paulo.

**Silveira LT, Barroso GM. 1999.** Revisão taxonomica do genero *Periandra* Mart. ex Benth. (Legumonosae, Papilionoideae, Phaseoleae). Revta Brasil. Bot., São Paulo, **22**(3): 339-356.

**Sinjushin AA. 2018.** Floral ontogeny in *Cordyla* *pinnata* (A. rich.) Milne-Redh. (Leguminosae, Papilionoideae): Away from stability. *Flora* 241: 8-15.

**Simpson BB. 1998.** A revision of *Pomaria* (Fabaceae) in North America. *Lundellia*, **1998**(1):46-72.

**Sinjushin AA. 2018.** Floral ontogeny in *Cordyla pinnata* (A. rich.) Milne-Redh. (Leguminosae, papilionoideae): Away from stability. *Flora*, **241**: 8-15.

**Sinjushin AA, Tekdal D, Ciftci C, Cetiner, S., 2018**. Floral development in *Thermopsis turcica*, an unusual multicarpellate papilionoid legume. *Plant systematics and evolution*, **304**(4): 461-471.

**Smitinand T, Larsen K. 1984.** *Flora of Thailand, Vol:4, Part: 1.* Royal Forest Department, Bangkok.

**Soepadmo E, Saw LG. 2000**. *Tree flora of Sabah and Sarawak, Vol: 3*. Forest Research Institute Malaysia.

**Sotuyo S, Contreras JL, Gagnon E, Lewis GP. 2017**. A synopsis of *Coulteria* (Leguminosae), including new names and synonyms. *Phytotaxa*, **291**(1): 33-42.

**Stace C. 2010.** *New flora of the British Isles*. Cambridge University Press.

**Stahl A. 1928.** *Estudios sobre la flora de Puerto Rico; con un prólogo de Carlos. E. Chardón*. Puerto Rico.

**Steyermark JA, Berry PE, Yatskievych K, Holst BK. 1995.** *Flora of the Venezuelan Guayana* *Vol. 4.* Saint Louis: Missouri Botanical Garden.

**Steyermark JA, Berry PE, Yatskievych K, Holst BK. 1995.** *Flora of the Venezuelan Guayana* *Vol. 5.* Saint Louis: Missouri Botanical Garden.

**Steyermark JA, Berry PE, Yatskievych K, Holst BK. 1995.** *Flora of the Venezuelan Guayana* *Vol. 6.* Saint Louis: Missouri Botanical Garden.

**Steyermark JA, Berry PE, Yatskievych K, Holst BK. 1995.** *Flora of the Venezuelan Guayana* *Vol. 8.* Saint Louis: Missouri Botanical Garden.

**Styer CH. 1977**. Comparative anatomy and systematics of Moutabeae (Polygalaceae). *Journal of the Arnold Arboretum*, **58**(2): 109-145.

**Thompson IR. 2010.** A Revision of *Cristonia* (Fabaceae: Brongniartieae). *Muelleria*, **28**(1): 66-73.

**Torrey J, Gray A. 1968.** *The* *Flora of North America:* Vol. 1. Hafner Publishing Company, New York.

**Townsend CC, Guest E. 1974.** *Flora of Iraq. vol. 3.* Bentham-Moxon Trust: Kew.

**Tucker SC. 1993.** Floral ontogeny in Sophoreae (Leguminosae: Papilionoideae). I. *Myroxylon* (*Myroxylon* group) and *Castanospermum* (*Angylocalyx* group). *American Journal of Botany*, **80**(1): 65-75.

**Tucker SC. 1994**. Floral ontogeny in Sophoreae (Leguminosae: Papilionoideae): II. Sophora sensu lato (*Sophora* group). *American Journal of Botany*, **81**(3): 368-380.

**Tucker SC. 2002**. Floral ontogeny in Sophoreae (Leguminosae: Papilionoideae). III. Radial symmetry and random petal aestivation in *Cadia purpurea*. *American journal of botany*, **89**(5): 48-757.

**Tucker SC. 2003**. Floral ontogeny in *Swartzia* (Leguminosae: Papilionoideae: Swartzieae): distribution and role of the ring meristem. *American Journal of Botany*, **90**(9): 1271-1292.

**Turner BL. 2008.** Revision of the genus *Orbexilum* (Fabaceae: Psoraleeae). *Lundellia*, **2008**(11): 1-8.

**Tucker SC. 1984.** Origin of symmetry in flowers. In: White RA and Dickison WC eds. Contemporary Problems in Plant Anatomy. Academic Press, Orlando, FL. 351-395.

**Tucker SC. 1990.** Loss of floral organs in *Ateleia* (Leguminosae: Papilionoideae: Sophoreae). Am J Bot. 77(6): 750-61.

**Tucker SC. 1992.** The role of floral development in studies of legume evolution. Can J Bot. 70(4): 692-700.

**Tucker SC. 1994.** Floral ontogeny in Sophoreae (Leguminosae: Papilionoideae): II. *Sophora sensu lato* (Sophora group). Am J Bot. 1994;81(3): 368-80.

**Tucker SC. 2000.** Evolutionary loss of sepals and/or petals in detarioid legume taxa *Aphanocalyx*, *Brachystegia*, and *Monopetalanthus* (Leguminosae: Caesalpinioideae). Am J Bot. 87(5): 608-24.

**Tucker SC. 2003.** Floral ontogeny in *Swartzia* (Leguminosae: Papilionoideae: Swartzieae): distribution and role of the ring meristem. Am J Bot.90(9): 1271-92.

**van der Niet T, Johnson SD. 2012.** Phylogenetic evidence for pollinator-driven diversification of angiosperms. Trends Ecol Evol. 27: 353-61.

**van Royen P, van Steenis CGGJ. 1952.** *Eriandra*, a new genus of Polygalaceae from New Guinea. *Journal of the Arnold Arboretum*, **33**(1): 91-95.

**van Steeins CGGJ, de Wilde WJJO. 1984.** *Flora Malesiana, Vol 10.* Kluwer Academic Publishers, Netherlands.

**van Steeins CGGJ, de Wilde WJJO. 1984.** *Flora Malesiana, Vol 12.* Kluwer Academic Publishers, Netherlands.

**Verdcourt B. 1978.** New taxa of Leguminosae from Papua New Guinea. *Kew Bulletin*,455-473.

**Warwick MC, Lewis GP, De Lima HC. 2008.** A reappraisal of *Barnebydendron* (Leguminosae: Caesalpinioideae: Detarieae). *Kew Bulletin*, **63**(1): 143-149.

**Webb CJ, Sykes WR, Garnock-Jones PJ. 1988.** Flora of New Zealand. Volume IV. PD Hasselberg, Government Printer

**Wendt T, Lott EJ. 1985**. A new simple-leaved species of *Recchia* (Simaroubaceae) from southeastern Mexico. *Brittonia*, **37**(2): 219-225.

**Wiggins IL. 1980.** *Flora of Baja California*. Stanford University Press.

**Wilkins C, Chappill J. 2007.** Three new species of *Latrobea* (Leguminosae: Mirbelieae) from south-western Australia. *Nuytsia*, **17**: 483-492.

**Wilson AJG. 1994.** *Flora of Australia, Volume 49 and 50, Oceanic Islands 1 and 2.* Australian Government Publishing Service, Canberra.

**Wojciechowski MF. 2003.** Reconstructing the phylogeny of legumes (Leguminosae): an early 21st century perspective. In: Klitgaard BB and Bruneau A eds. Advances in Legume Systematics, Part, 10, Royal Botanical Gardens, 5-35.

**Zarucchi JL. 1990.** A new species of *Macrolobium* (Fabaceae: Caesalpiniodeae) from Mesoamerica. *Annals of the Missouri Botanical Garden*, **77**(1): 209-211.

**Zhu XY. 2004,** January. A revision of the genus *Gueldenstaedtia* (Fabaceae). In *Annales Botanici Fennici*. Finnish Zoological and Botanical Publishing Board, 283-291.

Online Souces:

EFloras. 2008. Published on the Internet [http://www.efloras.org](http://www.efloras.org/) [accessed May 2018] Missouri Botanical Garden, St. Louis, MO & Harvard University Herbaria, Cambridge, MA.

<https://pfaf.org/user/Default.aspx>

<https://davesgarden.com/guides/pf/>

<http://rubens-plantasdobrasil.blogspot.com/search/label/>

<https://www.delta-intkey.com/www/data.htm>

<https://plantfacts.osu.edu/pdf/0247-1047.pdf>

<http://swbiodiversity.org/seinet/taxa/index.php?taxon=1521>

http://floranorthamerica.org/files/Surianaceae05a%20Gal%20web.pdf

<https://biogeodb.stri.si.edu/biodiversity/taxa/22303?filter_order=24356>

<http://pza.sanbi.org/plants/search/advanced?pow_page=family&name=Fabaceae&title_sort=ASC>

Thailentadopsis Kostermans (Leguminosae: Mimosoideae: Ingeae) Resurrected<https://alchetron.com/Parapiptadenia-rigida>

<https://www.monaconatureencyclopedia.com/arthroclianthus-deplanchei/?lang=en>

<https://www.fpl.fs.fed.us/documnts/TechSheets/Chudnoff/TropAmerican/html_files/amburanew1.html>

<https://www.rhs.org.uk/Plants/156323/Argyrocytisus-battandieri/Details>

<https://www.burncoose.co.uk/site/plants.cfm?pl_id=5393>

<http://www.tropicalforages.info/key/forages/Media/Html/entities/cratylia_argentea.htm>

<http://www.aridzonetrees.com/pithecellobium-pallens.html>

<https://apps.cals.arizona.edu/arboretum/taxon.aspx?id=393>

<http://www.public.asu.edu/~camartin/plants/Plant%20html%20files/ebenopsisebano.html>

<https://garden.org/plants/view/203954/Poincianella-mexicana/>

<http://nzpcn.org.nz/flora_details.aspx?ID=181>

<https://www.feedipedia.org/node/292>

<https://www.nndfw.org/nnhp/Plants/erro.pdf>

https://www.mozambiqueflora.com/speciesdata/

<https://wnmu.edu/academic/nspages/gilaflora/fabaceae.html>

<https://www.gardenersworld.com/plants/>

<https://www.wildflower.org/plants/result.php?id_plant=STAF4>

<https://florabase.dpaw.wa.gov.au/>

<http://sweetgum.nybg.org/science/world-flora/>

https://raskisimani.files.wordpress.com/2013/01/inocarpus-tahitian-chestnut.pdf

<http://www.ville-ge.ch/musinfo/bd/cjb/africa/details.php?langue=an&id=69985>

<http://yabanicicekler.com/home>

<https://florafaunaweb.nparks.gov.sg/special-pages/plant-detail.aspx?id=3027>

<http://tropical.theferns.info/>

<http://plantnet.rbgsyd.nsw.gov.au/>

<http://www.kyffhauser.co.za/species.htm>

<https://www.brit.org/sites/default/files/public/BRIT%20Press/IFNCT_Docs/FNCT_0618-1076-Fab-Zygo.pdf>

http://www.pngplants.org/

[http://www.plantsoftheworldonline.org/](http://www.plantsoftheworldonline.org/taxon/urn:lsid:ipni.org:names:23567-1)

<http://copperflora.org/eflora/index.php>

<http://www.onlineplantguide.com/Index.aspx>

<https://hort.ifas.ufl.edu/database/documents/pdf/tree_fact_sheets/lysbaha.pdf>

<https://edis.ifas.ufl.edu/pdffiles/ST/ST36600.pdf>

<https://pdfs.semanticscholar.org/87eb/b80b683105b927c9b9a37cf441fe219a7cc7.pdf>

<https://uses.plantnet-project.org/en/Main_Page>

<https://www.prota4u.org/database/>

<http://portal.cybertaxonomy.org/flora-malesiana/>

<http://gateway.myspecies.info/>

<https://plants.jstor.org/compilation/mildbraediodendron.excelsum>

<https://dpipwe.tas.gov.au/Documents/Stonesiella-selaginoides.pdf>

<http://keys.trin.org.au/>

<https://www.anbg.gov.au/cpbr/cd-keys/peakey/key/The%20Pea%20Key/Media/Html/Thinicola.html>

<https://wetlandinfo.des.qld.gov.au/wetlands/ecology/components/flora/>

<http://www.illinoiswildflowers.info/>

<http://powo.science.kew.org/taxon/urn:lsid:ipni.org:names:23840-1>

<https://www.tropicalgrasslands.asn.au/>

<https://www.zambiaflora.com/speciesdata/index.php>

<http://www.wildflower.org/plants/result.php?id_plant=EBEB>
